# Geographic Information Systems, spatial analysis, and HIV in Africa: A scoping review

**DOI:** 10.1371/journal.pone.0216388

**Published:** 2019-05-03

**Authors:** Danielle C. Boyda, Samuel B. Holzman, Amanda Berman, M. Kathyrn Grabowski, Larry W. Chang

**Affiliations:** 1 Department of International Health, Bloomberg School of Public Health, Johns Hopkins University, Baltimore, MD, United States of America; 2 Division of Infectious Diseases, Department of Medicine, Johns Hopkins University School of Medicine, Baltimore, MD, United States of America; 3 Johns Hopkins Bloomberg School of Public Health Center for Communication Programs, Baltimore, MD, United States of America; 4 Department of Pathology, Department of Medicine, Johns Hopkins University School of Medicine, Baltimore, Maryland, United States of America; University of the Witwatersrand, SOUTH AFRICA

## Abstract

**Introduction:**

Geographic Information Systems (GIS) and spatial analysis are emerging tools for global health, but it is unclear to what extent they have been applied to HIV research in Africa. To help inform researchers and program implementers, this scoping review documents the range and depth of published HIV-related GIS and spatial analysis research studies conducted in Africa.

**Methods:**

A systematic literature search for articles related to GIS and spatial analysis was conducted through PubMed, EMBASE, and Web of Science databases. Using pre-specified inclusion criteria, articles were screened and key data were abstracted. Grounded, inductive analysis was conducted to organize studies into meaningful thematic areas.

**Results and discussion:**

The search returned 773 unique articles, of which 65 were included in the final review. 15 different countries were represented. Over half of the included studies were published after 2014. Articles were categorized into the following non-mutually exclusive themes: (a) HIV geography, (b) HIV risk factors, and (c) HIV service implementation. Studies demonstrated a broad range of GIS and spatial analysis applications including characterizing geographic distribution of HIV, evaluating risk factors for HIV, and assessing and improving access to HIV care services.

**Conclusions:**

GIS and spatial analysis have been widely applied to HIV-related research in Africa. The current literature reveals a diversity of themes and methodologies and a relatively young, but rapidly growing, evidence base.

## Introduction

Geographic Information Systems (GIS) are systems designed to store, manage and display spatial data and aid in analysis and interpretation of spatial data [1, 2]. Spatial analysis are techniques applied to geographic data to measure, interpret, and explore characteristics and associations [1–4]. Geographic data can include defining the locations of a point in space (known as point pattern data), quantifying values of a characteristic at a given location (geostatistical data), or describing an entire geographical region (area-level data) [1, 5–7]. With the rise and increasing accessibility of computing power and relevant software, GIS and spatial analysis are finding wider audiences and novel applications [3, 8–10].

Notably, GIS and spatial analysis can be powerful tools to understand, prevent, and help treat diseases. For example, as a visualization aid, GIS software can be used to map the geographic distribution of disease, associated risk factors, and services available for prevention and treatment. Furthermore, spatial analysis of this data can analyze risks for disease, epidemic trends over space and time, and disease hotspots [9, 11]. Taken together, these tools can contribute to the design, planning, and allocation of global health resources for prevention and treatment services, as well as help to assess intervention impact.

HIV continues to be a major global health threat, particularly in Africa which accounts for 67% of the world’s infections, highlighting a need for additional approaches to understand and mitigate the epidemic [12]. GIS and spatial analysis techniques have begun to be applied to HIV-related research in sub-Saharan Africa. However, it is unclear to what extent and depth these tools have been utilized. In this scoping review focused on providing an overview of a broad topic [13], we sought to systematically find and inductively summarize the literature on applications of GIS and spatial analysis to HIV-related research in Africa. Our goal is to help inform researchers, implementers, and policy makers on how GIS and spatial analysis tools have been used, and to explore underlying themes and methodologies.

## Methods

### Eligibility criteria

#### Inclusion criteria

This review included peer-reviewed articles that focused on HIV in Africa and involved GIS or spatial analysis. HIV-related topics included HIV prevalence or incidence, HIV-specific mortality, HIV risk factors, and implementation of HIV prevention or treatment services. Only articles explicitly based in a country or countries in Africa were included. Spatial analysis techniques and the GIS use were defined broadly for the purposes of this review in order to capture the diversity of emerging practices, i.e. we included articles that used any specialized GIS software or specifically incorporated any spatial analysis techniques. Articles were included regardless of participant characteristics, including age, race, sex, sexual orientation, HIV status or other factors. Articles were included regardless of sample size, scope or setting within Africa, type of program activities or interventions, or by study design.

#### Exclusion criteria

We excluded studies concerning non-opportunistic infection health outcomes in HIV-positive individuals or for which HIV infection was not the main focus but treated as a risk factor. We did not include studies that used geographic coordinates solely for study enrollment purposes, e.g to generate sampling frames, or basic visualization, e.g. simple display of points on a map. We excluded HIV phylogenetic studies, unless they involved other spatial analyses, on the basis that such research is complex and specialized enough to warrant its own review. Abstracts, posters, reviews and commentary pieces were also excluded.

#### Information sources and search

We developed a search strategy with terms relating to “HIV”, “geographic information systems”, and “spatial analysis” (see Appendix for full search strategy). This search was developed through an iterative process of incorporating new terms and refining those included based on results returned and identification of relevant citations. Reviewers conducted electronic searches of PubMed, EMBASE, and Web of Science on November 16, 2017, with no restriction on date or language of publication. Potentially eligible articles known to paper authors were also included in the initial search.

#### Study identification and data collection

Two reviewers independently screened article titles and abstracts of all initial search results. Articles deemed eligible for inclusion by either reviewer underwent full text review by three authors to determine final inclusion with adjudication done by majority vote when necessary. Reviewers extracted data to a master table, capturing details about location, objectives, study design, data source and study population, spatial analysis methodologies, software used, and results.

#### Synthesis of results

Following data extraction, we categorized studies based on the primary content of the paper in an inductive, thematic analysis. Articles were permitted to sort into more than one category as relevant. We did not assess study quality due to study heterogeneity.

## Results

### Study selection

**Fig 1:**
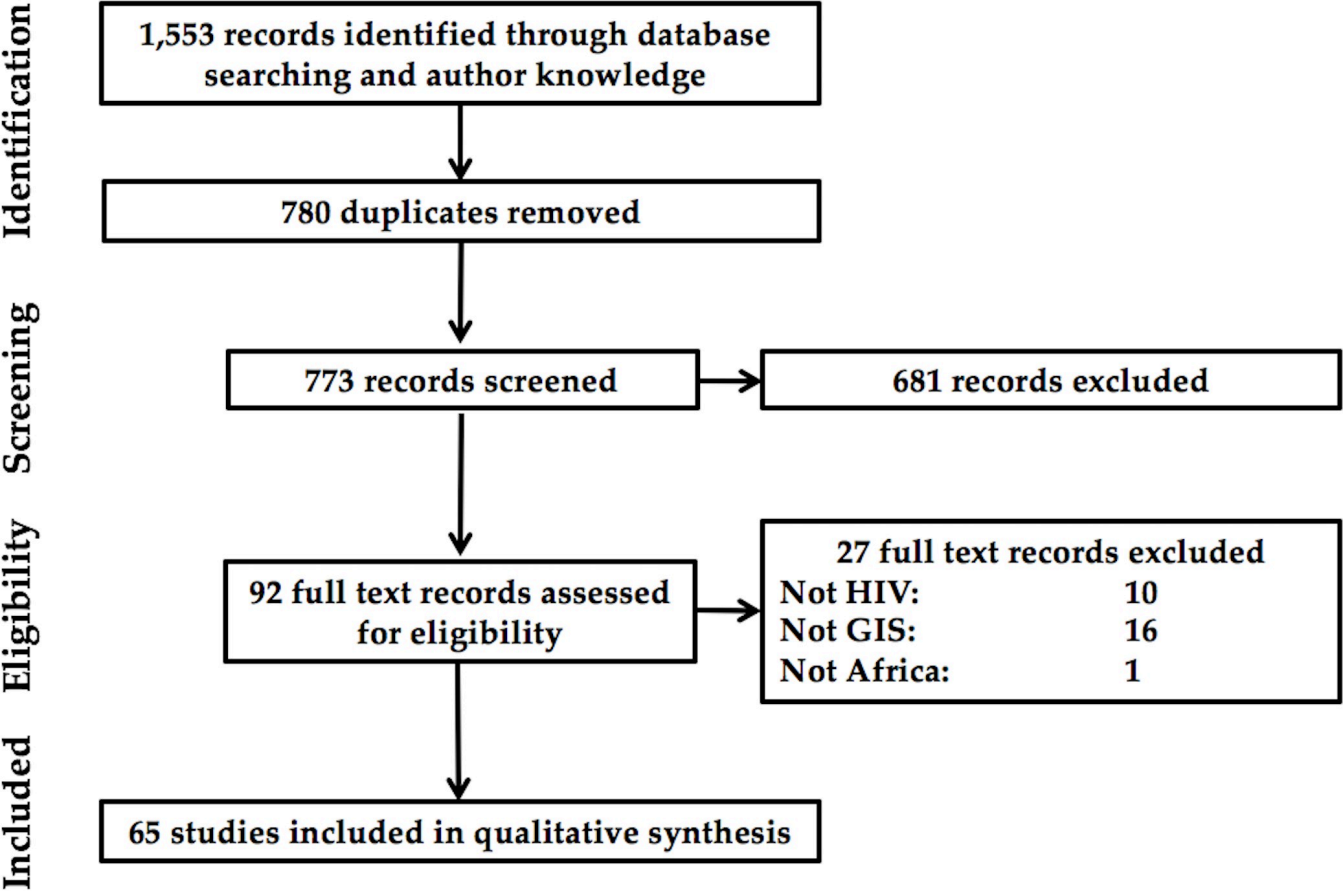
Flowchart of article selection.

As shown in Fig 1, 773 references were identified, excluding 780 duplicates, of which 681 were screened out as not meeting eligibility requirements. Full text review of the remaining 92 studies identified 65 final articles for inclusion in the review. The reasons for exclusion were that the study was not based in Africa (1 reference), that the study was not primarily focused on HIV-related topics (10 references), and that the analysis did not involve spatial analysis (16 references).

Included studies spanned the continent in geographic focus. Eight papers were multi-country analyses, and 15 different countries were represented in single-country papers. Among single-country studies, 37 were based in Southern Africa, 16 in East Africa, and two each in West Africa and Central Africa. South Africa was the most common country of study with 17 papers. Dates of publication spanned from 2000 to 2017, with almost all (98%) published between 2006–2017 and over half published after 2014.

### Study themes

Inductive thematic analysis found that GIS and spatial analysis were most commonly used to examine: (a) HIV geography, (b) HIV risk factors, and (c) HIV service implementation. We discuss methodological techniques in each theme and narratively and in tabular format highlight key findings below.

### HIV spatial epidemiology

Twenty-seven articles sorted into this thematic category which focused on the spatial distribution of HIV. The following subcategories were identified and used to further characterize studies: (i) spatial cluster analysis, and (ii) smoothed mapping.

#### Spatial clustering analysis

One of the most common spatial analytic techniques for evaluating the geographic distribution of HIV was local cluster detection (Table 1), or the identification of HIV disease “hotspots”. Clusters are areas with higher or lower numbers of events in a particular study region than expected if cases are randomly distributed [14].

**Table 1:** Studies using cluster detection and clustering analysis to characterize the spatial distribution of HIV.

AUTHOR	COUNTRY	ANALYSIS	SIZE	KEY FINDINGS
**BARANKANIRA ET AL. [18]**	Burundi	Kulldorff cluster detection to describe spatial variation of HIV prevalence.	8,086	One high and one low HIV cluster, all independent of provincial boundaries.
**CHIMOYI & MUSENGE [21]**	Uganda	Kulldorff cluster detection for HIV prevalence.	7,518	One significant primary and 15 tertiary clusters that highlight Central and Eastern regions as most at-risk.
**CUADROS ET AL. [16]**	20 countries	Kulldorff cluster detection of high and low HIV. Evaluate association of national HIV prevalence with population size and strength of cluster(s).	20 countries	Low prevalence countries had stronger clusters of high HIV prevalence. High prevalence countries had stronger clusters of low HIV prevalence.
**CUADROS & ABU-RADDAD [17]**	Cameroon, Ethiopia, Kenya, Lesotho, Malawi, Mali, Rwanda, Senegal, Tanzania, Zimbabwe	Compare change in HIV prevalence within high-HIV Kulldorff clusters vs. outside of high-HIV clusters.	10 countries	HIV prevalence within high-prevalence clusters either did not decline or increased, even if national prevalence declined.
**CUADROS & ABU-RADDAD [28]**	Cameroon, Kenya, Lesotho, Tanzania, Malawi, Zambia, Zimbabwe	Kulldorff cluster detection of sero-discordant couples and high HIV prevalence.	16,140	No spatial pattern for sero-discordancy independent of HIV prevalence patterns. HIV prevalence correlated with proportion of couples that were sero-discordant.
**GONZÁLEZ ET AL. [22]**	Mozambique	Kulldorff cluster detection to compare HIV over time.	722 (2010), 789 (2012)	Small cluster of high HIV in 2010 persisted and grew in 2012.
**GRABOWSKI ET AL. [31]**	Uganda	Clustering analysis to determine the likelihood that a participant living in the same household as an HIV-positive person, or within given distance rings from an HIV-positive person would also have HIV.	14,594	Strong clustering within households: sharing a household with an HIV-positive person increased likelihood of HIV by 3.2. Weaker clustering within 10-250m (1.2 times likelihood) and 250-500m (1.08 times likelihood).
**LAKEW ET AL. [20]**	Ethiopia	Kulldorff cluster detection for HIV prevalence.	30,625	Two clusters and spatial heterogeneity identified.
**MEE ET AL. [26]**	South Africa	Space-time Kulldorff cluster detection for HIV/TB mortality and non-HIV/TB mortality during decentralization of ART provision.	73,000	Two low-risk and one high-risk HIV/TB mortality clusters detected. Unclear link to ART decentralization.
**MESSINA ET AL. [19]**	Democratic Republic of Congo	Kulldorff cluster detection of HIV by sex.	9,755	Detected clusters of HIV with evidence that spatial distribution and intensity varies by sex.
**NAMOSHA ET AL. [25]**	South Africa	Kulldorff cluster detection for HIV mortality comparing pre- and post-ART roll-out.	86,175	Strong clusters persisted over time. High-mortality clusters in peri-urban communities near National Road.
**SARTORIUS [27]**	South Africa	Comparison of identified high-mortality and low-mortality Kulldorff clusters.	1,110,166 person-years	Identified clusters and several risk factors that differed significantly between high and low clusters.
**SCHAEFER ET AL. [24]**	Zimbabwe	Compare HIV service uptake and demographic characteristics inside and out of identified HIV Kulldorff clusters.	8,092	Two high-prevalence and one low-prevalence clusters of HIV. High HIV clusters were urban, wealthier, and had better access but less uptake of HIV services.
**TANSER ET AL. [23]**	South Africa	Compare characteristics inside and out of identified HIV Kulldorff clusters.	12,221	High and low clusters detected. Settlements near National Road had highest prevalence. High prevalence communities have high education, household wealth, employment, lower marriage and migrants.
**ZULU ET AL. [29]**	Malawi	Global and local Moran’s I and Getis-Ord Gi* statistics to identify district-level clusters and outliers for high and low HIV prevalence over 8 time periods.	54 ANCs	Identified hotspots and coldspots that moved somewhat and shrank over time.

Thirteen studies used the Kulldorff spatial scan statistic [15] to identify clustering of HIV infection, [16–24], HIV-specific and all-cause mortality [25–27], and sero-discordant partnerships [28]. In these studies, the spatial scan statistic identified statistically significant “hotspots” or “cold spots” by systematically scanning circular windows of varying sizes across the study area, comparing the number of cases within the window to those outside the window. The resulting data were used to identify areas of particular risk or concentrated need, and to compare characteristics inside and outside of clusters. SaTScan software was the most commonly used tool for identifying clusters (see S1 Table for software used in each study).

Clusters of high and/or low HIV prevalence were detected, independent of administrative boundaries, in 20 of 22 countries where the Kulldorff spatial scan statistic was applied, though the geographic size and relative risk strength of the clusters varied widely. Studies that compared clusters over time found that they tended to persist with little change in location [17, 22, 25]. In one study, areas contained within high-prevalence clusters had stable or increasing HIV prevalence over time, even when HIV significantly decreased in the rest of the country [17].

Less commonly-used spatial statistics included Local Moran’s I and the τ statistic [29]. Both of these methods are global clustering statistics which measure the tendency for points to occur closer together in space by chance across the entire study area. In contrast, the Kulldorff spatial scan statistic identifies local clusters in a particular region. Local clusters can exist in either the absence or presence of global clustering [30]. In HIV epidemiology, global clustering statistics have been used to determine if HIV-positive people tend to live closer to each other than would be expected by chance. For example, a study in Uganda conducted a global clustering analysis and [31] found that a participant living in the same household as an HIV-positive person was 3.2 times more likely to be HIV-positive than any study participant; however, the authors found no significant spatial clustering of cases outside the household.

#### Smoothed mapping of HIV

Smoothed maps, or “heat maps” of HIV use spatial methods to create and display continuous gradations of HIV-related outcomes over space, using data gathered in a limited number of locations to predict values in unmeasured locations (Table 2). Eighteen studies in this review created smoothed maps of HIV [17–19, 23, 24, 27–29, 32–41]. Methods for creating smoothed maps of HIV varied in complexity. Simpler methods were based on calculating weighted averages of existing data surrounding an unmeasured location. These included inverse distance weighted estimates (IDW) [17, 19, 28, 29, 34, 38] and kernel density smoothing with a fixed [18, 23] or varying radius [32, 33]. More complex methods, such as kriging or Bayesian hierarchical modeling with a spatial component, used statistical models to predict HIV prevalence [24, 27, 32, 34–37, 39–41]. Software use varied by method, with IDW and kriging maps typically created using ArcGIS [38], kernel density smoothing done in BayesX [18] or Idrisi [23], and Bayesian modeling done in R [35, 36], WinBUGS [39, 40], or OpenBUGS [27] (see S1 Table).

**Table 2:** Studies creating continuous surface maps of HIV.

AUTHOR	COUNTRY	TYPE OF SPATIAL PREDICTION	SIZE	KEY FINDINGS
**BARANKANIRA ET AL. [18] **	Burundi	**Kernel density smoothing**	8,086	Spatial heterogeneity independent of administrative boundaries. Identified locations in need of HIV resources.
**CARREL ET AL. [35]**	Democratic Republic of Congo	**Bayesian kriging** of 2007 and 2013 HIV data, subtracting the maps to show areas of greatest difference.	9275 (2007), 18,257 (2013)	HIV prevalence decreased in urban locations and increased in rural locations, but areas of high difference were relatively small.
**CHANG ET AL. [41]**	Uganda	**Bayesian modeling** of percent and number of people living with HIV (PLHIV) per km^2^	17,119	High HIV prevalence along Lake Victoria and patchy prevalence in district interior. Areas with highest number of PLHIV were inland in high population-density trading centers.
**COBURN ET AL. [38]**	Lesotho	**Inverse distance weighted (IDW)** mapping combined with population density map to display the number of HIV-positive persons per km^2^.	7,099	Density of infection is significantly higher in urban areas, but the majority of HIV-positive people live dispersed in rural areas.
**CUADROS & ABU-RADDAD [17]**	Cameroon, Ethiopia, Kenya, Lesotho, Malawi, Mali, Rwanda, Senegal, Tanzania, Zimbabwe	**IDW** mapping for visualizing differences in spatial distribution of HIV between time periods.	10 countries	HIV prevalence within high-prevalence clusters either did not decline or increased, even if national prevalence declined.
**CUADROS ET AL. [37]**	Tanzania	**Kriging** of HIV prevalence and male circumcision rates to assess their spatial correlation.	2003–04: 12,522 2007–08: 16,318 2011–12: 18,809	Areas of low male circumcision overlap with areas high HIV prevalence, and vice versa.
**CUADROS & ABU-RADDAD [28]**	Cameroon, Kenya, Lesotho, Tanzania, Malawi, Zambia, Zimbabwe	**IDW** mapping of HIV and sero-discordancy prevalence to assess their spatial correlation.	16,140	No spatial pattern for sero-discordancy independent of HIV prevalence patterns.
**KALIPENI & ZULU [34]**	Continental	**IDW** and **kriging** interpolation of international HIV prevalence for country-level estimates. Model epidemic curves for each country and project future trends.	1,442 sentinel sites over 18 years	Differences between UNAIDS estimates vs. kriging- and IDW-generated national estimates were statistically insignificant. Nearly all countries have reached maturity level of epidemic curve.
**KLEINSCHMIDT ET AL. [36]**	South Africa	**Bayesian kriging** map of HIV prevalence among youth.	11,758	Variation in HIV prevalence independent of provincial boundaries, highest in the east and for women.
**LARMARANGE & BENDAUD [33]**	17 countries	**Kernel density estimation** with adaptive bandwidths (prevR) to generate sub-national HIV estimates.		Continuity of HIV estimates across borders. Certainty of estimates varied depending on total sampling size, total number of administrative units, distribution of survey clusters across area.
**MESSINA ET AL. [19]**	Democratic Republic of Congo	**IDW** HIV prevalence maps by sex to create regional-level estimates.	9,755	Spatial variation in HIV, distribution and intensity varied by sex.
**SARTORIUS ET AL. [40]**	South Africa	**Bayesian kriging** of all-cause and cause-specific child mortality risk.	46,675	Two geographic foci of high mortality, matching areas of high HIV/TB mortality.
**SARTORIUS ET AL. [39]**	South Africa	**Bayesian kriging** of all-cause and cause-specific adult mortality risk.	104,969	Five geographic foci of high mortality, correlating to areas of high HIV/TB mortality.
**SARTORIUS [27]**	South Africa	**Bayesian kriging** of age-specific all-cause and HIV/TB mortality risk.	1,110,166 person-years	Spatial distribution of all-cause mortality risk varied by age group, reflecting spatial trends in HIV/TB mortality.
**SCHAEFER ET AL. [24]**	Zimbabwe	**Kriging** of HIV prevalence and uptake of HIV testing and counseling (HTC).	8,092	HIV prevalence higher in two urban areas for men and women, but HTC uptake lower in those areas and in one other.
**SUBNATIONAL ESTIMATES WORKING GROUP [32]**	Tanzania, Kenya, Malawi	**Comparison of six methods.** Pixel-level estimates: - Kernel density estimation with adaptive bandwidths (prevR) - Bayesian model-based geostatistics - Kriging of each covariate with regression to combine of layers Administrative unit-level estimates: - Shared spatial component model - Regression kriging at aggregated scale - Bayesian geo-additive mixed model.		All methods revealed within-country variations and were similar in accuracy, but Bayesian geostatistical approach slightly better.
**TANSER ET AL. [23]**	South Africa	**Kernel density smoothing** to estimate spatial distribution of HIV.	12,221	Spatial variation in HIV prevalence with highest prevalence in urban settlements near the National Road.
**ZULU ET AL. [29]**	Malawi	**IDW** HIV prevalence maps for eight years to compare trends over time.	19 ANCs for time trends	Spatial variation independent of district boundaries, shifting spatial patterns over time.
**GONESE ET AL. [44] **	Zimbabwe	**Compare** ANC surveillance with geographically proximate DHS data.	7,202 (ANC) 13,049 (DHS)	ANC and DHS similar for most populations, but ANC estimates were lower for women within 30km of ANC site.
**MUSINGUZI ET AL. [43]**	Uganda	**Compare** HIV prevalence rates between ANC surveillance sites and national population survey clusters within 30km.	16,936 (UHSBS); 9,668 (ANC)	Overall estimate similar. ANC-based was higher in ages 15–19, lower for those aged 30+, and in urban areas.

Two studies compared different mapping techniques, and the most comprehensive study found that a Bayesian geostatistical modeling approach was slightly more accurate than five other methods [32, 34]. However, it was also noted that the choice of mapping method may have less impact on the accuracy of the HIV estimates than the underlying quality and sampling frame of the data used [32–34]. HIV estimates derived from smoothed maps and aggregated to the country level were found to align closely with more complex UNAIDS model data, regardless of mapping methodology [34].

Smoothed mapping techniques generated maps which estimate HIV prevalence in areas where data is not available from surveys [17, 29, 32–34, 42]. Studies that generated smoothed maps found high-risk areas that stretched across more than one administrative area [19, 29, 32–34, 36, 37], or high prevalence pockets in provinces that would not necessarily have been identified as high priority settings [29, 32, 36]. As Kleinschmidt et al note, a high prevalence area that they detected across two South African provinces could benefit from inter-provincial coordination of treatment and prevention service provision [36].

Detailed smoothed maps also enabled visual comparisons of the spatial patterns of HIV over time periods [17, 29, 34, 35], or between various subgroups of the population, such as men and women or those living in sero-discordant partnerships and those not [19, 28, 36, 39]. GIS software also helped validate HIV prevalence estimates from antenatal care surveillance against population-based survey estimates, which are more comprehensive, but more resource-intensive, by comparing data between the two sources at similar locations [43, 44].

### HIV risk factor analysis

Thirty-three studies sorted into this thematic subcategory which focused on the spatial epidemiology of HIV which is based on the concept that both HIV and the factors associated with infection vary spatially and have some degree of spatial dependence. Articles in this theme divided into three sub-topics based on underlying methods utilized: (i) spatial regression, (ii) joint spatial disease/risk factor modeling, and (iii) geography of risk factors.

#### Spatial regression

Spatial analysis contributed to the evaluation of risk factors for HIV by testing the assumption that data is independent of geography (Table 3). Many studies noted that this independence assumption was violated if data displayed spatial dependence or correlation, meaning that data points in close proximity to each other were more alike than those further apart [18, 21, 27, 40, 45–50]. To address this issue, thirteen studies included in their analysis spatial regressions with spatial random effects to represent the correlations within geographic areas and between geographically proximate areas [18, 21, 27, 39, 40, 45–52]. These analyses used BayesX [18, 21, 45, 46, 48–50], WinBUGS [27, 39, 47]and OpenBUGS Bayesian software [40]. Studies that compared non-spatial and spatial regressions found that the spatial models had better fit [45–47]. Spatial models may have been more accurate in these studies because they accounted for underlying, unmeasured environmental, social, behavioral or other confounding factors relevant to HIV prevalence that were geographically determined.

**Table 3:** Studies performing spatial regressions, regressions with spatially varying coefficients, and joint spatial modeling.

AUTHOR	COUNTRY	METHODOLOGY	SIZE	OUTCOME OF INTEREST	UNIT Spatial effects	KEY FINDINGS
**SPATIAL REGRESSIONS**						
**BARANKANIRA** **ET AL [18]**	Burundi	Bayesian spatial logistic regression	8,086	Factors associated with HIV after controlling for spatial heterogeneity	Province-level	After controlling for spatial variation, HIV associated with female sex, older age, marital status, higher wealth index, sexual history, 12-month STI history, and higher education level.
**CHIMOYI AND** **MUSENGE [21]**	Uganda	Bayesian spatial binomial logistic regression compared with non-spatial regression	7,518	Factors associated with HIV before and after controlling for spatial heterogeneity	Region-level	Spatial effects influenced distribution of HIV after adjusting for demographic and social/behavioral factors. Factors that influenced HIV in the non-spatial model were not significant after adjusting for spatial variation.
**DOCQUIER ET** **AL [52]**	44 countries	Dynamic Spatial Error and Spatial Auto-Regressive models	44	Spread of HIV across country borders	Country-level	Emigration to high-prevalence destinations associated with origin country's HIV prevalence. Insignificant spatial correlation suggests that emigration accounts for spatial variation.
**KANDALA ET** **AL [49]**	Zambia	Bayesian geo-additive spatial regression	3,950	Geographic distribution of HIV	Province-level	After controlling for spatial variation and age, the two highest prevalence provinces were no longer among the areas with highest HIV.
**KANDALA ET** **AL [50]**	Zambia	Bayesian geo-additive spatial regression	5000 (2001), 11,138 (2007)	Change in geographic distribution of HIV over 6 years	Province-level	Two regions changed from low to high-risk or high to low-risk over 6 years. Adjusting for spatial variation changed the HIV risk of two provinces in each time period.
**KANDALA ET** **AL [48]**	Botswana	Bayesian geo-additive spatial regression	15,878	Geographic distribution of HIV	District-level	Highest HIV prevalence along the Zimbabwe border after controlling for demographic and social/behavioral factors
**MUSENGE ET** **AL [46]**	South Africa	Bayesian spatial zero inflated negative binomial regression	16,844	Risk factors for child HIV/TB mortality	Household-level	Three mortality hotspots. Nine significant demographic and social factors after controlling for spatial variation.
**MUSENGE ET** **AL [45]**	South Africa	Bayesian spatial logit regression	6,692	Geographic distribution of child HIV/TB mortality	Household-level	High mortality hotspot with higher maternal deaths, male child mortality and lack of health facility access.
**NGESA ET AL** **[47]**	Kenya	Bayesian geo-additive spatial regression	3,662	Geographic distribution of HIV	County-level	Highest HIV prevalence in the western part of Kenya around Lake Victoria after controlling for demographic and social/behavioral factors.
**SARTORIUS** **ET AL [40]**	South Africa	Bayesian spatial Cox proportional hazards regression	46,675	Space-time variation in child mortality	Village level	Main cause of mortality is HIV/TB and mortality increased over time. Two hotspots of mortality identified. Multiple individual- and household-level risk factors after controlling for spatial variation.
**SARTORIUS** **ET AL [39]**	South Africa	Bayesian spatial Weibull parametric regression	104,969	Space-time variation in adult mortality	Village-level	Main cause of mortality is HIV/TB. Five hotspots of mortality identified. Mortality increased over time until 2008 with numerous individual-, household-, and community-level risk factors after controlling for spatial variation.
**SARTORIUS** **[27]**	South Africa	Bayesian spatial negative binomial and Weibull parametric regressions	1,110,166 person-years	Space-time variation in age-specific mortality	Village-level	Multiple, differing hotspots of mortality, temporal trends and social/behavioral risk factors identified for each age group after controlling for spatial variation.
**WIRTH ET AL** **[51]**	Botswana	Pairwise composite likelihood approach for spatially-correlated binary data	6,745	Geographic distribution of HIV	Sextile bands of geographic distance from HIV hotspot	HIV prevalence significantly lower in 3rd, 4th and 6th sextile of distance away from HIV hotspot.
SPATIALLY VARYING COEFFICIENTS AND JOINT DISEASE MODELLING						
**MANDA ET AL** **[54]**	South Africa	Bayesian spatial joint modeling regression	101,472	Geographic distribution and correlation of HIV and syphilis	District-level	HIV and syphilis negatively correlated across space. Geographic concentrations of each disease more apparent after controlling for risk factors.
**OKANGO ET** **AL [55]**	Kenya	Non-spatial regression, Bayesian spatial joint modeling regressions	4,864	Geographic distribution and correlation of HIV and HSV-2	County-level	Spatial model had best fit. HIV and HSV-2 significantly spatially correlated, with higher risk of both infections in regions around Lake Victoria in the west of the country.
**OKANGO ET** **AL [53]**	Kenya	Bayesian spatially varying coefficients regression	4,864	Geographic variation in the effect of risk factors on HIV and HSV-2	County-level	Risk factor variation across space was significant for HSV-2 but not for HIV. Visually, the effects of some demographic and social factors for HIV were stronger in some counties than others.
**WABIRI ET AL** **[56]**	South Africa	Non-spatial regressions (with Moran's I for residuals) and geographically weighted regressions (GWR) for demographic and for social covariates	15,000	Geographic variation in the effect of risk factors on HIV	District-level	GWR model was a better fit and non-spatial regressions had significant spatial correlation in residuals. Hyper-epidemic districts have homogenous populations of black Africans, high proportion single or with partner 5+ years older.

Seven studies used the results of spatial regression to map adjusted relative HIV risk over geographic areas, controlling for other factors like age, sex and employment. Maps of adjusted HIV risk visualized spatial patterns and heterogeneity, displaying relative risk by administrative area [21, 47–50] or over smoothed, continuous surfaces [45, 46]. In Zambia, spatial patterns of HIV changed after adjusting for age and spatial correlation so that the two highest-prevalence districts were no longer among the areas at highest risk [49]. These results, together with similar findings elsewhere [48–50], highlight the extent to which geographic location can provide insights on complex or poorly understood risks for HIV that would not be captured in a standard statistical model.

#### Joint spatial disease/risk factor modeling

Four studies examined how the relationship between HIV and a risk factor or other sexually transmitted infection (STI) changed over geographic space (Table 3) [53–56]. Such spatial variation may have been due to unmeasured, underlying modifying or cofounding factors that varied across space, such as attitudes, cultures and preferences, or due to sampling variation [53]. Spatial analysis was used to evaluate and display how different risk factors [53, 56] or other STIs [53–55] were more or less correlated with HIV across different geographic areas. Joint spatial modeling was conducted with WinBUGS [54, 55], R [53]or a combination of ArcGIS and STATA software [56].

These studies aimed to assist resource allocation decisions by identifying opportunities for geographic targeting of HIV prevention interventions [53, 56]. They searched for geographic areas where particular risk factors were more relevant to HIV outcomes, for instance finding regions where age at first sex had a stronger association with HIV prevalence [53]. However, models that allowed the effect of each risk factor to differ by spatial location were not consistently better or worse fitting than models that held relationships constant over space [53, 56]. There was some evidence that the impact of social risk factors relating to sexual habits varied more over space than the impact of demographic risk factors in a South African analysis [56].

Three studies compared spatial patterns of HIV and another STI, either HSV-2 [53, 55] or syphilis [54], in order to address questions of population-level ecological associations between STIs. These studies examined the extent to which these STIs were spatially correlated, whether because the infections were mutual risk factors or because they had similar underlying, potentially unmeasured causes in Africa [53–55]. Joint spatial modeling revealed that syphilis prevalence did not predict HIV prevalence in South Africa, given their low degree of spatial correlation, and as such would not be suitable as a proxy measure to examine ecological differences in risky sexual behavior resulting in HIV infection [54].

#### Geography of HIV risk factors

GIS enabled the systematic measurement of spatial risk factors (Table 4). For example, GIS technology allowed for the characterization of a defined area around a study participant’s community, such as the risk of schistosomiasis [57] or the density of refugee camps within a 25km radius [19]. Similarly, GIS tools were used to measure distances between a household’s location and the nearest city [19, 35], major road [19, 58], body of water or refugee camp [19]. One study used geo-referenced mobile phone data from a cell service provider in Côte d’Ivoire to develop a predictive model of area HIV prevalence based on residents' travel and communication patterns [42].

**Table 4:** Geography of risk.

AUTHOR	COUNTRY	RISK FACTOR	METHODOLOGY	SIZE	KEY FINDINGS
**COVARIATE(S) MEASURED WITH GIS**					
**BRDAR ET AL [42]**	Côte d’Ivoire	Mobile phone usage data relating to social connectivity, spatial location, migration and movement, and activity.	Predictive Ridge and Support Vector regression models	5 million mobile phone users	Night-time connectivity and activity, area covered by users and overall migrations are strongly linked to HIV prevalence. Models based on spatial features were highly predictive of HIV.
**BRODISH AND SINGH [57]**	Mozambique	S. haematobium exposure (distance to high-endemic areas)	Regression analysis	8,847	Exposure to S. haematobium increased the odds of HIV by three times, controlling for demographic and sexual risk factors.
**CARREL ET AL [35]**	Democratic Republic of Congo	Distance to the nearest city	Poisson mixed effects regression comparing two time periods	9275 (2007), 18,257 (2013)	Urban HIV prevalence decreased and rural HIV increased between 2007 and 2013. Protective effect of distance to city disappeared.
**MESSINA ET AL [19]**	Democratic Republic of Congo	Distance to cities, rivers, refugee camps, conflict sites	Regression analysis	9,755	Proximity to city and distance to river (for women) associated with HIV.
**TANSER ET AL [58]**	South Africa	Mean distance from household to major road	Regression analysis	16,583	Distance to major road strongly correlated with HIV prevalence.
**ZULU ET AL [29]**	Malawi	Distance/time to roads, public transport and health facilities, proximity to cities, and elevation	Regression analysis and mapping of clusters and outliers of selected risk factors relative to HIV prevalence (local Moran's I and Getis-Ord Gi*)	54 ANCs for risk analysis	Mean travel time to public transport for ages 30–44 associated with HIV. Distance to main road protective. Hotspots and coldspots of relationship between risk factors and HIV identified in different areas.
**SPATIAL ANALYSIS OF RISK FACTORS**					
**ABIODUN ET AL [64]**	Nigeria	Early sexual debut	Bayesian spatial Cox hazards model for spatial analysis of early sexual debut.	4,301	Northern states significantly earlier sexual debut after controlling for other factors.
**AKULLIAN ET AL [65]**	Kenya	HIV stigma	Describe spatial patterns of HIV stigma using difference of K-function cluster analysis and spatial regression.	373	Spatial trend and clustering in external stigma (blame) but not internal stigma (shame).
**AKULLIAN ET AL [60]**	Kenya	Male circumcision	Smoothed map of circumcision in 2008 and 2014.	484 (2008); 1649 (2014)	Clear boundary in circumcision prevalence between traditionally circumcising areas in 2008, diminished in 2014 after VMMC program implementation.
**CUADROS ET AL [63]**	Kenya, Malawi, Tanzania	Malaria	Smoothed map (model-based geostatistics) of malaria prevalence to calculate covariate in logistic regression.	19,735	People living in high malaria prevalence areas were nearly twice as likely to be HIV positive as those living in low malaria areas.
**CUADROS ET AL [37]**	Tanzania	Male circumcision	Compare Kuldorff clusters and LISA hotspots of male circumcision (MC) and HIV. Compare HIV incidence by gender inside and outside MC cold spots.	2003–04: 12,522; 2007–08: 16,318; 2011–12: 18,809	Outside of low-MC clusters, females at greater risk than males, but inside low-MC clusters, males and females at equal risk.
**CUADROS AND ABU-RADDAD [28]**	Cameroon, Kenya, Lesotho, Tanzania, Malawi, Zambia, Zimbabwe	Sero-discordant partnerships	Compare Kuldorff clusters of sero-discordant couples and HIV prevalence. Compare epidemiologic measures of discordancy inside and outside clusters.	16,140	No spatial pattern for sero-discordancy independent of HIV prevalence patterns. HIV prevalence correlated with proportion of couples that were sero-discordant.
**PALK AND BLOWER [62]**	Lesotho	Couples with one member temporarily living away from home	Kriging maps of divided household by absent member (husband vs. wife) and their temporary residence (within country vs. South Africa). Regression on HIV status and extramarital partnerships.	2,026 couples	Spatial patterns of divided households differed based on where the absent partner was. No significant association between divided household and HIV. Absent wives increased the risk of extramarital partners for men.
**SARTORIUS [27]**	South Africa	Clusters of age-specific mortality	Comparing Kuldorff clusters of high and low mortality rates	1,110,166 person-years	Multiple social and demographic characteristics identified that significantly differed between high and low mortality clusters
**TANSER ET AL [23]**	South Africa	Clusters of high and low HIV	Compare characteristics of high and low HIV clusters.	12,221	High prevalence clusters have high education, household wealth, employment, lower marriage and migrants.
**WAND AND RAMJEE [61]**	South Africa	Education, age at sexual debut, cohabitation with partner, number of recent partners, transactional sex	Geo-additive spatial regression of risk factors and HIV risk at two clinics.	3,462	Women at Botha's Hill clinic had higher education, more sexual partners and less marriage. Total risk score showed higher impact on Botha's Hill women than Umkomaas.
**WESTERCAMP ET AL [59]**	Kenya	Sexual behaviors and STI history	Kuldorff cluster detection for STIs and sexual behaviors among young men	649	No clusters detected other than condom use.
**ZULU ET AL [29]**	Malawi	Distance to main roads, travel time to public transport, ever having tested for HIV, education, syphilis	Mapping of clusters and outliers of selected risk factors relative to HIV prevalence (local Moran's I and Getis-Ord Gi*)	19 ANCs for time trends; 54 ANCs for risk analysis	Hotspots and coldspots of each explanatory variable relative to HIV identified in different areas.

Local cluster detection methods were used to identify clusters of HIV risk factors, for instance of condom non-use [59]. These risk factor clusters anticipated areas that might benefit from prevention services, and were compared to mapped clusters of HIV to display the overlap between risk and outcome [29, 37]. Two studies compared incidence of new HIV infections inside and outside of risk factor clusters [28, 37]. Three of the studies that identified clusters of high HIV prevalence [23, 28] or mortality [27] also compared characteristics inside and outside of these clusters to identify associated risk factors.

Smoothed maps of risk factors showed geographic variation independent of administrative boundaries enabling new insights [28, 37, 60–62]. Smoothed mapping of malaria endemicity enabled the estimation of malaria prevalence at every point where HIV prevalence had been measured [63]. Studies compared the spatial distribution of a risk factor to the spatial distribution of HIV to assess evidence of geographic concurrence [28, 37, 57, 61]. Geographic overlap of particular risk factors and HIV enabled a community- or population-level analysis of their relationship, rather than individual-level analysis, and highlighted the extent to which location influences the HIV epidemic.

Risk factors were also examined with spatial regression methods, such as early sexual initiation [64] or HIV stigma [65]. In Nigeria, for example, controlling for spatial correlations, demographic and knowledge covariates, and the non-linear effect of age revealed a clear North-South dichotomy in early sexual initiation [64]. Studies analyzing the geography of risk factors often used GIS software like ArcGIS [19, 28, 29, 63], Idrisi [58], and SaTScan [23, 59] to extract spatial data about the risk factor that was then incorporated into an analysis in statistical software like R, STATA, SPSS, and others. Alternatively, some analyses were conducted entirely in R [35, 60–62, 65], BayesX [64] or STATA, using thefunctions in these software applications to handle both spatial and statistical analyses, such as the geonear function in STATA [57].

### HIV services implementation

Twenty-three articles contributed to understanding relationships between geography and implementation of HIV services. Two subcategories were the (i) effect of distance to HIV services on health outcomes, and (ii) planning and evaluating HIV service provision.

#### Effect of distance

GIS technology allowed for measurement of geographic distances and distribution of services and enabled analysis of the effect of distance on health outcomes and service uptake (Table 5). For example, GIS tools enabled the calculation of distances between points, including both Euclidean straight-line distance [29, 40, 58, 66–72] and road distance [73–76] between households and clinics, providing objective measures of geographic burdens to health care access. Evidence was mixed on whether these measures were [77] or were not [66, 75, 78] correlated with self-reported measures of health care barriers. Studies also examined the impact of proximity to health care on presence of HIV risk hotspots [45, 69] and HIV clusters [24, 70, 72, 79].

**Table 5:** Studies regarding the effect of distance to health care.

AUTHOR	COUNTRY	ACCESS BARRIERS	OUTCOME	SIZE	TYPE OF ANALYSIS	FINDINGS
**AKULLIAN ET AL. [66]**	Uganda	Distance, time, cost of travel to facility	Healthcare access	379	Regression	PLHIV travel further for care than non-PLHIV.
**BASSETT ET AL. [73]**	South Africa	Distance to testing site	HIV testing	4,701	Compare testing for HIV at mobile vs clinic-based sites	Mobile testers more likely to test <1km or >5km from home than fixed-site testers.
**BUEHLER ET AL. [72]**	Mozambique	Distance to ARV clinic	HIV knowledge	3749	Clusters of high or low knowledge (Getis-Ord statistic) relative to clinic locations. Regression.	Clustering of higher HIV knowledge closer to facilities. Distance negatively associated with outcome.
**CARLUCCI ET AL. [77]**	Zambia	Distance, road distance, travel time to clinic	ARV adherence	424	Regression	Measures of distance correlated with each other but not associated with outcome.
**CLOUSE ET AL. [80]**	South Africa	Distance between clinic where woman initiated ART and clinic where she re-entered care	Time to re-entry, CD4 count at re-entry	300	Comparison of median and IQR values between women re-entering care in the same province vs. a different province	Post-partum women who re-entered care in a different province had a higher median distance to their new facility, re-entered care faster and had better CD4 count outcomes.
**COOKE ET AL. [68]**	South Africa	Distance to clinic	ARV initiation	1,660	Regression	Distance negatively associated with outcome.
**ESCAMILLA ET AL. [69]**	Zambia	Distance to clinic	PMTCT uptake	254	Uptake density (kernel density estimates) in relation to clinic locations. Regression.	Areas with high-density uptake were located near health centers. Distance negatively associated with outcome, with a 1.9km threshold.
**GOLUB ET AL. [67]**	Kenya	Distance to male circumcision facility	Male circumcision follow-up	1437	Regression	Distance negatively associated with outcome for fixed facilities but not mobile facilities.
**HOUBEN ET AL. [76]**	Malawi	Travel time to ART clinic based on smoothed map of least travel time from each point	Accessing ART at nearest clinic; transferring clinics	5,411	Comparison of estimated and actual travel time between two time periods. Regression.	Travel time and transfers declined, uptake increased as ART clinics opened. Proportion of patients not attending nearest clinic increased slightly.
**JOHNSON ET AL. [71]**	Malawi	Distance from neighborhood to clinic	Timely ARV initiation	15,734	Regression	Distance negatively associated with outcome for one clinic but not the other, located next to central transport hub.
**MEE ET AL. [74]**	South Africa	Distance to clinic	Biomedical vs traditional health use	2,833	Visual analysis of outcome in relation to clinics. Regression.	No spatial patterns or significant association.
**MUSENGE ET AL. [45]**	South Africa	Health facility presence, minimum distance to clinic	HIV/TB mortality	6,692	Bayesian spatial regression with visual analysis of odds ratio map in relation to clinic locations.	Odds ratio hotspot is area furthest from clinics. Distance covariate not significant.
**SIEDNER ET AL. [78]**	Uganda	Distance and route distance, travel time and cost to clinic	HIV clinic attendance	188	Regression	GPS distances negatively associated with outcome, self-reported measures not associated.
**SARTORIUS ET AL. [40]**	South Africa	Distance to clinic	All-cause and HIV/TB mortality	46,675	Regression	Distance not significantly associated with outcome.
**SCHAEFER ET AL. [24]**	Zimbabwe	Distance to clinic	HIV testing	8,092	Compared distance and uptake inside and outside high and low clusters of HIV prevalence (Kuldorff spatial scan)	Distance not associated with outcome. Those living in high-prevalence clusters had better access but lower uptake of HIV testing.
**TANSER ET AL. [58]**	South Africa	Distance to road	HIV prevalence	2,013	Regression	Distance negatively associated with outcome.
**YAO ET AL. [70]**	Mozambique	Distance to clinic, # clinics within distance radii	HIV testing	1025	Clusters of high and low testing (Kuldorff spatial scan) in relation to clinic locations at three time periods. Regression.	Clusters of high testing tended to be near testing clinics and clusters of low testing tended to be far. Distance to testing clinic negatively associated with outcome.
**YAO ET AL. [79]**	Mozambique	Distance to clinic	HIV testing	1680	Clustering (K-function, Kuldorff spatial scan) and spatial dependence (Moran's I, LISA) of testing. Regression.	Clustering and spatial dependence observed but no patterns relative to clinics. Distance to clinic negatively associated with outcome.
**ZACHARIAH ET AL. [75]**	Malawi	Road distance, transport costs to hospital	ARV initiation	740	Regression	Cost negatively associated with outcome, road distance not associated.
**ZULU ET AL. [29]**	Malawi	Distance to clinic, distance to road, time to public transportation	HIV prevalence	54 ANCs	Regression	Distance to road negatively associated with outcome, time to public transportation for ages 30–44 positively associated.

Studies identified in this review analyzed the effect of distance to care on nearly all stages of the HIV treatment cascade, including knowledge about HIV [72], follow-up visits for male circumcision prevention services [67], HIV testing and counseling [24, 70, 73, 79], healthcare attendance [66, 74, 78], ARV uptake [68, 71, 75, 76] and adherence [77, 80], PMTCT uptake [69], and HIV mortality [29, 40, 45]. The majority of studies found that proximity to health care was associated with positive health outcomes at each step of the cascade [45, 66–72, 76, 78, 79], though these findings were not universal [24, 40, 71, 74, 75, 77].

In some studies, access for HIV patients appeared to be mediated through the public transportation network more than through road access [29, 71, 75], particularly for patients who were further away from the clinic [71]. This relationship may reflect the positive correlation between road transportation and HIV transmission observed in some settings [29, 58]. There was furthermore evidence that the effect of distance on service access differed between those accessing mobile care compared with those attending static clinics [67, 73]. HIV-positive patients did not necessarily transfer to a closer clinic when given the chance [66, 76], and postpartum women who switched clinics returned to care faster and with better outcomes when they transferred to distant clinics than when they re-entered nearby clinics [80].

Distance measures were extracted with GIS software like ArcGIS [26, 29, 60, 67, 70, 72–74, 76, 77, 79, 81], ArcMap [80], MapInfo [68], Google Earth [71], Idrisi [23] and associations were analyzed in a standard statistical software such as STATA, R and SPSS (see S1 Table for more details).

#### Service provision

GIS technology was used to examine the extent to which health care service expansion [60, 70, 82] and outreach to communities [68, 73, 76] increased access and uptake (Table 6). Decentralization of HIV services was found to dramatically decrease the average distance traveled for ART among people living with HIV [68, 76] and to diminish the variation in service uptake over space [60, 70]. For example, as HIV testing became more widely available in Mozambique, clusters of high or low rates of testing disappeared, indicating that the geographic barriers to testing became less relevant [70].

**Table 6:** Studies relating to service provision.

AUTHOR	COUNTRY	OBJECTIVES	SIZE	KEY FINDINGS
**AKULLIAN ET AL. [60]**	Kenya	Smoothed map of circumcision in 2008 and 2014.	484 (2008); 1649 (2014)	Clear boundary in circumcision prevalence between traditionally circumcising areas in 2008, diminished in 2014 after VMMC program implementation.
**BASSETT ET AL. [73]**	South Africa	Compare the yield, geographic distribution and demographics of mobile vs clinic-based HIV testing services.	5327	Mobile testers differed from clinic testers in age, gender, and distance travelled to test. HIV prevalence at mobile sites differed by type of venue.
**COBURN ET AL. [38]**	Lesotho	Map the density of HIV infection to compare coverage across districts under efficient vs. equitable resource allocation.	7099	Majority of HIV-positive people live in low-density rural areas that would receive low coverage in optimally efficient resource allocation. Coverage would range from 4% to 94% if areas with 5 infected people per km^2^ (70% national coverage) were prioritized.
**COOKE ET AL. [68]**	South Africa	Comparison of median distance traveled for ART over time.	7576	Median distance decreased from 34.2km to 3.1km when treatment was made available through all primary healthcare facilities.
**HOUBEN ET AL. [76]**	Malawi	Track changes in travel time to the nearest clinic providing ART and clinic actually attended as services expanded between 2005 and 2009.	5411	Median travel time to the nearest and attended clinics fell, uptake increased, and the proportion not attending their nearest ART clinic increased slightly.
**YAO AND MURRAY [82]**	Mozambique	Compare current and optimized allocation of HIV testing sites to minimize population-weighted travel distances. Evaluate efficiency gains of adding or relocating services to new locations.	53 clinics	Optimization of 2009 services would improve average access distance by 24.4%. Clinics chosen for expanded or relocated services in areas of low testing rates. Optimization would relocate 12 clinics or expand to 11 new clinics.
**YAO ET AL. [70]**	Mozambique	Assess impact of expanding HIV services on access to and use of HIV testing with regression analysis and Kuldorff cluster detection.	1025	Decentralization of services reduced variation in testing rates

Two studies used GIS tools to target HIV services and improve their efficiency [38, 82]. One study mapped the density of HIV infection in Lesotho in order to calculate the geographic variation of coverage if the country was to optimize efficiency in treating 70% of its HIV-positive population [38]. In rural areas where most of the HIV-positive population lives, the authors found, coverage would be as low as 4%, whereas the densely populated urban areas would receive up to 94% coverage. Yao and Murray [82] used GIS technology along with optimization software Gurobi to optimize the location of HIV testing and counseling sites, so that the overall distance traveled by the entire population to their nearest testing site would be as short as possible. Software like ArcGIS [38, 70, 73, 76], ArcView [60]and MapInfo [68]was used for visualization and extraction of distance data to be analyzed in statistical software like STATA and R.

## Discussion

The review demonstrates the wide applications of GIS and spatial analysis to understanding HIV and supporting HIV care and prevention services in Africa. Most of the literature was published recently and rapid growth in the evidence-base was clear. We expect that GIS use and spatial analysis methodologies will continue to expand as more researchers and implementers develop knowledge and capacity in this growing field and increasingly recognize its usefulness.

For example, in this review GIS tools were regularly used to display HIV prevalence, incidence or mortality data in order to communicate complex information about the epidemic in a clear and accessible way. The ability to identify spatial clusters and generate smoothed maps revealed variations within and across administrative regions, the boundaries of which often have little bearing on disease prevalence.

In the context of HIV risk factor analysis, spatial analysis reflected the likelihood that people living close to each other share common exposures and disease outcomes and that location was critical in including in HIV risk factor analyses. The findings that spatial regressions of HIV which accounted for geographic correlation in the data had better fit than non-spatial regressions highlight the benefit of adding spatial data to certain analyses.

Another recurring theme was the critical role of geography in informing access to HIV-related health services in low-income settings with poor existing health care infrastructure. GIS and spatial analysis may allow for more efficient allocation of resources and appropriate response targeting in many African settings.

Of note, while this review was focused on more technologically and methodologically sophisticated applications, several articles not included in this review demonstrated the utility of simply visually displaying mapped data points, such as for plotting the locations of HIV services [83] and risky sexual behaviors [84–88]. Mapping the overlap of service provision and demand was useful for selecting priority areas for programmatic expansion [81, 89, 90]. Program implementation could benefit from dynamic, interactive and iteratively updated maps with verified health facility coordinates and infrastructure data [91] and simple mapping may be good entry point for more advanced geospatial techniques.

A number of gaps in the literature were also found by this review. West African countries were less represented, perhaps because they have lower HIV prevalence rates and less HIV research capacity. Central African and Horn countries were similarly less represented. Countries that did not include both HIV biomarkers and geographical coordinates in their DHS surveys also may be less likely to feature in spatial analysis studies because these were characteristics that determined inclusion in certain studies. Some important HIV related outcomes had minimal or no found spatial studies such as HIV medication shortages or stock-outs. Adherence and retention outcomes were also not substantially considered in relation to spatial factors. Finally, there was limited literature on HIV resistance and spatial analysis, potentially because these data are too sparse.

Interesting directions and issues in spatial research were also found. The novel use of cell phone technology in some studies points to an important future area of spatial research. Data independent of national boundaries/national data collection practices to facilitate cross-country comparisons and multi-country analyses would be a helpful future direction given the mobility of persons and complexity of social networks. Privacy concerns continue to be an important area of consideration as GIS tools become easier to use and more broadly implemented.

### Limitations

Our search strategy may have missed studies relating to GIS or spatial analysis and HIV, particularly as the vocabulary for GIS is dispersed and evolving. There is some risk of publication bias, potentially favoring articles that detect spatial heterogeneity over those that do not, or favoring particular spatial analysis methods over others. This review was not intended to assess the quality, methodological rigor or risk of bias within studies included for review, nor did we attempt to conduct a meta-analysis. In part, this decision reflects the wide diversity of contexts, approaches, and methodologies that emerged through the review, which were difficult to synthesize. There are not currently any best practice or guidelines for reporting of geospatial studies, and this may be an area for future development.

## Conclusions

This systematic review searched for and summarized evidence on the use of GIS and spatial analysis techniques for HIV in Africa. Our findings demonstrate the wide array of spatial approaches to HIV-related data. These applications include characterizing geographic distribution of HIV, evaluating HIV epidemiologic risk factors, and assessing and improving implementation of HIV services. The rapid growth and diversity of applications of GIS and spatial analysis to the field of HIV yields great potential for future insights and progress.

## Supporting information

S1 Table All articles included in review. (DOCX)

S1 File Search term details. (DOCX)

S2 File PRISMA 2009 checklist. (DOC)
