# Supplementary material for: Geographic Information Systems, spatial analysis, and HIV in Africa: A scoping review
Source: PLoS One. 2019 May 3;14(5):e0216388. doi: 10.1371/journal.pone.0216388 (PMC6499437; doi:10.1371/journal.pone.0216388)
Supplement: S1 Table — (DOCX) [file pone.0216388.s001.docx]

| **Authors** | **Country** | **Objectives** | **Software** | **Covariates** | **Results** |
| --- | --- | --- | --- | --- | --- |
| Abiodun et al. (2014) | Nigeria | Identify factors associated with early sexual debut among youth, including spatial patterns at the state level that persist after controlling for various other factors, using a Bayesian Cox hazards model | BayesX | Sex, age, education, urban/rural, religion, knowledge of STIs and HIV/AIDS, state of residence | Controlling for covariates and ethnic group/census block random effects changed the states with earlier than expected sexual debut so that it was mostly a North/South dichotomy with Northern states significantly associated with early sexual debut and Southern states significantly associated with delayed debut. Fixed effects for later sexual initiation: Males (HR: 0.69), cohort of 20-24yo's (HR: 0.79), higher education compared to no education (HR: 0.58, similar values for all ed levels above none), urban (HR: 0.81). Census blocks accounted for more unobserved heterogeneity than ethnic group. |
| Akullian et al. (2014) | Kenya | Describe spatial variation in HIV stigma using cluster analysis and spatial regression | ArcView 10.0 for visualization, R version 3.0.2 for spatial data analyses | Age, household income, education, occupation, marital status, number of mobile phones in household, self-reported HIV status, knows someone with HIV | More clusters for those without external stigma than for those with external stigma. Association between internal vs. external stigma is modified by HIV status. Large-scale spatial trend in external stigma after adjusting for other factors (p<0.01) No spatial trends in internal stigma (p = 0.70). |
| Akullian et al. (2016) | Uganda | Compare healthcare travel burdens and facility choices among PLHIV and non-PLHIV with regression analysis | ArcGIS 10.1 | Age, sex, household income, size of household, education, marital status, occupation, mode of transportation, distance to nearest clinic, distance to clinic accessed, cost of travel to clinic | PLHIV traveled 1.9km further (p=0.004) than those not living with HIV to access care. No evidence that PLHIV traveled further than the nearest health facility if that facility offered ART (p=0.328), but if it didn’t, they were 56% less likely to access care at nearest facility (p=0.011). PLHIV tended to seek care at a higher tiered facility and traveled an extra 2.2km (p=0.001) than people not living with HIV to access high tier facility. Self-reported cost and travel time not significant. |
| Akullian et al. (2017) | Kenya | Compare smoothed map of circumcision in 2008 and 2014. | R 3.2.2 | No covariates included in the spatial visualization using GAM except for a random effect for the enumeration area. Age, county, urban/rural, marital status, ethnicity, education, self-reported sexual risk behavior. | Circumcision prevalence increased from 45.6% (2008) to 71.4% (2014). Increases in circumcision prevalence particularly in young men and men in areas with lower prevalence in 2008. 2008 maps show boundary between areas with traditional circumcision and without, but 2014 map for 15-24 y.o. has only smaller areas with lower circumcision rather than a boundary. |
| Barankanira et al. (2016) | Burundi | Describe spatial variation of HIV with mapping and cluster analysis. Identify associations with demographic factors with spatial and non-spatial regressions | STATA 11, R, SaTScan version 9.3, BayesX version 2.1 | Sex, age, education, marital status, religion, wealth index, sexual activity, extramarital sex partners, STI history, province | Moran's I = 0.03, p=0.021. Spatial heterogeneity of HIV prevalence ranging 1-10%. Clusters: high prevalence cluster around Bujumbura-Mairie (RR = 3.7, p<0.001); low prevalence in south (no cases, p=0.02). Multivariate spatial analysis: HIV associated with female sex (OR = 1.36, p=0.001), older age (OR = 1.97, p=0.004), marital status (OR 1.86, p-0.004), higher wealth index (OR: 2.11 p<0.001), sexual activity (OR: 1.76, p = 0.034), history of STI in last 12 months (OR = 2.03, p<0.001), education (OR = 1.5, p<0.001). |
| Bassett et al. (2015) | South Africa | Compare people testing for HIV in mobile vs. clinic-based testing sites. Analyze data from mobile HIV testing program to optimize deployment | ArcGIS | Sex, age, HIV status, distance between household and testing site | Mobile testers: more likely to be male (p=0.002), younger (p=0.002), HIV-negative (p<0.001), not to get CD4 count tested if HIV-positive (p<0.001) or to return for results (p<0.001). Prevalence at mobile testing sites varied by type of venue: taxi stands and commercial sites had highest prevalence (17-26%). People who tested furthest from home tested at largest commercial center. 64% testers lived closer to clinic than mobile testing site. Mobile testers more likely than clinic testers to be tested within 1km of residence, but also more likely than clinic testers to be tested 5+km from home (p<0.001) |
| Brdar et al. (2015) | Côte d'Ivoire | Develop predictive models for HIV prevalence based on cell phone records capturing communication and mobility patterns | QGIS for maps, R for HIV prevalence; not reported for predictive models | Communication flow between cell phone antennas (number of calls) of each department; geographic location of users (home department and travel to other departments): radius of gyration, area and perimeter of convex hull of users' movements, diameter of range, night communication; long-term mobility (longer than 3 days); time and day of communication and travel | Communication hubs correspond to HIV hot spots; larger hubs have higher HIV prevalence. Mobility flows connect between geographically close departments and between two largest cities, connecting regions with higher prevalence. Abidjan is hub of long-term migrations, has highest prevalence and high connectivity. Predictive modeling: SVR model with RFE performed best, though performance declined when departments with poor HIV estimates were included. Best result for all departments was SVR model including subset of activity features (correlation coefficient = 0.627, RRMS = 0.509). Night-time connectivity and activity, area covered by users and overall migrations are strongly linked to HIV prevalence. Models based on spatial features (gyration, area, perimeter, diameter and distance) highly predictive (correlation coeff = 0.753, RRMSE = 0.294). |
| Brodish & Singh (2016) | Mozambique | Assess relationship between HIV risk and S. haematobium exposure (unimproved water source and proximity to high-endemic point) in women using regression analysis | STATA | Age, urban/rural, education, wealth index, marital status, religion, >2 lifetime sexual partners, geographical zones around high-endemic schistosomiasis points | Areas around high-endemic schistosomiasis points have more HIV cases, though a higher percentage of people reporting improved water source were HIV positive. Map of high HIV clusters and coordinate points of high S haemotobium prevalence shows that sites of likely coinfection tend to be on or near rivers. Some evidence from map of geographic overlap between higher female:male HIV ratio and areas of likely coinfection. |
| Buehler et al. (2015) | Mozambique | Map hotspots of high and low HIV knowledge and evaluate associations between knowledge and demographic factors, including distance to nearest ARV clinic, with regression analysis | ArcGIS 10.2; R 3.0.2 | Age, education, household size, # children under 5, numeracy, literacy, district, distance to nearest ART facility, HIV knowledge score | Some significant clustering of higher and lower HIV knowledge (p<0.05); Age, higher education, increasing household size, proximity to health facility correlated with higher HIV knowledge; Non-linear relationship between distance and knowledge (5km vs >35km from HIV clinic = half point higher on 10-point scale of knowledge) |
| Carlucci et al. (2008) | Zambia | Evaluate the effect of travel to care on ART adherence using regression analysis | ArcGIS 9.1 | Age, gender, education, linear distance, actual distance, travel time, mode of transport, cost of transport, perceived stigma, WHO stage at initial visit, BMI at initial visit, days of adherence, days on ART | Multivariable regression: Days observed on ART significantly associated with optimal adherence (OR:1.04 per day, p=0.002). Travel duration, WHO stage, BMI, perceived stigma, cost of transport all not significantly associated with adherence. Nor were linear or actual distance significant. Travel duration correlated with linear and actual distance (Spearman rank correlation = 0.56 p<0.0001) |
| Carrel et al. (2016) | Democratic Republic of Congo | Determine whether HIV has diffused from cities into rural settings using Bayesian kriging and Poisson mixed effects regression | R 3.0.2 | Sex, year of survey, distance of survey cluster to nearest city, interaction between year and distance | Urban HIV prevalence decreased and rural HIV increased between 2007 and 2013, though changes were mostly isolated to small pockets of central, south east and northeast DRC. In 2007, effect of increased distance was 0.996 but in 2013, effect of distance was 0.999. Interaction term statistically significant at 5% level. |
| Chang et al. (2016) | Uganda | Describe geographic distribution of HIV prevalence and number of people living with HIV (PLHIV) per sq km using Bayesian modeling | Stan 2.8 | Sexually active adults, multiple sex partners, non-marital partners, alcohol before sex, partner outside the community, genital ulcer disease, condom use, ART use and male circumcision. Age and sex-controlled. | HIV prevalence ranged from 9% to 43% across communities, with higher HIV prevalence (41%) in fishing communities. Trading communities = 17% and agrarian communities = 14%. In fishing communities, ART coverage (15%) and male circumcision (18%) lower and risk behaviors higher for men than women. Smoothed map of HIV prevalence showed high prevalence along Lake Victoria and patchy prevalence in the interior with high and low prevalence areas next to each other. Highest prevalence near Lake Victoria, highest number of PLHIV in higher-density major trading centers inland. |
| Chimoyi & Musenge (2014) | Uganda | Identify HIV clusters using cluster detection. Evaluate regional variation and other risk factors associated with HIV in youth aged 15-24 using spatial and non-spatial regression analysis | STATA 12, Bayes X, SaTScan 9.0, Quantum GIS | Education, marital status, circumcision, transactional sex, multiple sexual partners, condom use, STI presence, alcohol use, age at sexual debut | Cluster detection: one primary and fifteen secondary clusters. Primary cluster in Central and Eastern regions (OR: 2.14) with tertiary clusters in north-east, east and south-east. Spatial binomial regression: Factors associated with high HIV included previous marriage (POR: 3.23, p<0.01), sex while inebriated (POR: 1.32, p=0.02), presence of STI in last 12 months (POR: 1.72, p<0.01). Factors protective: marriage (POR: 0.39, p<0.01), circumcision (POR: 0.64, p<0.01), condom use (POR: 0.54, p<0.01). |
| Clouse et al. (2017) | South Africa | Comparison of median and IQR distance values from initial clinic to destination clinic between women re-entering care in the same province vs. a different province | ArcMap; database: TherapyEdge-HIV, SAS 9.4 | Age at ART initiation, CD4 count at ART initiation, nationality, employment status, education | Lost to follow up dropped from 38.1% to 25% when women who went to a different clinic were counted. Most women switched to a nearby clinic, 1/3 switched to a clinic in a different province. |
| Coburn et al. (2017) | Lesotho | IDW mapping combined with population density map to display the density of HIV-positive persons in order to compare coverage across districts under efficient vs. equitable resource allocation. | ArcGIS (with ArcMap Advanced 10.1 software), R. | None. | The majority of HIV-infected persons live in rural areas with low density of infection. Allocating treatment as prevention resources for optimal cost-efficiency would concentrate on high-density urban areas with at least 5 PLVIH per square km. Variations in coverage by district: up to 94% in urbanized districts, down to 4% in rural districts. |
| Cooke et al. (2010) | South Africa | Evaluate factors associated with ART initiation, including distance to clinic, using regression analysis | STATA 10; Mapinfo 2.0 | Age, sex, education, wealth index, urban/rural, distance to clinic | Median distance to ART was 34.2km when only available at hospital vs. 8.5km when available in community health facility vs. 3.1km when available in PHC clinics. Multivariable regression: age was significant predictor of uptake (highest odds in 50-54 age group), distance to the nearest health facility significant predictor of uptake: likelihood of accessing ART decreased 27% for each square-root transformed km to facility. At 4.78km from clinic, odds of uptake 50% of odds for someone living next door. |
| Cuadros & Abu-Raddad (2014) | Cameroon, Ethiopia, Kenya, Lesotho, Malawi, Mali, Rwanda, Senegal, Tanzania, Zimbabwe | Identify variation in decline of HIV prevalence by comparing change in prevalence within high-HIV clusters vs. outside of high-HIV clusters | ArcGIS 9 for IDW; cluster detection not specified | None. | Clusters detected in four countries: Tanzania, Malawi, Kenya and Zimbabwe. HIV prevalence within high-prevalence clusters either didn't decline or increased even if national prevalence declined. Tanzania: national prevalence -27% (p<0.001), no decline in high HIV clusters (p=0.14), one cluster increased (p=0.01). Malawi: national prevalence -12% (p=0.09), no decline in cluster (p=0.76). Kenya: insignificant national decline -6% (p=0.62), cluster prevalence +20.1% (p=0.01). Zimbabwe: national prevalence -16.7% (p<0.001), cluster increased +18.5% (p<0.001). No clear differences in demographics between in-cluster and out-of-cluster -- clusters more urban for Malawi, Tanzania and Zimbabwe, but less urban for Kenya |
| Cuadros & Abu-Raddad (2016) | Cameroon, Kenya, Lesotho, Tanzania, Malawi, Zambia, Zimbabwe | Identify clusters of sero-discordant couples and of high HIV prevalence using cluster detection. Evaluate associations with sero-discordant partnerships using correlation coefficient | SatScan 9.4.2, SAS 9.3, ArcGIS 9.2 | None. | Spatial clusters of sero-discordant prevalence detected in Kenya, Malawi, Tanzania, mostly overlapping with high HIV prevalence. Proportion of sero-discordant couples out of all couples significantly higher in high HIV clusters (except in Cameroon and Lesotho). Proportion of sero-discordant couples out of HIV-affected couples not significantly different inside or outside of clusters. Proportion of individuals in sero-discordant couples out of all individuals were significantly higher in clusters in Tanzania, Kenya, Malawi and Zambia, compared to those outside of clusters. Proportion of HIV-positive individuals in sero-discordant partnerships significantly lower in clusters in Malawi, Zambia, Zimbabwe, and Lesotho, compared to those outside of clusters. Positive correlation between HIV prevalence and proportion of sero-discordant couples out of all couples (PSS = 0.89) and between HIV prevalence and proportion of individuals in sero-discordant couples out of all individuals (PSS = 0.6). |
| Cuadros et al. (2011) | Kenya, Malawi, Tanzania | Smoothed map (model-based geostatistics) of malaria prevalence to calculate covariate in logistic regression of malaria endemicity on HIV prevalence. | ArcGIS 9.2, R 2.11.1 | Age, urban/rural, education, religion, marital status, wealth index, presence of genital ulcerations during the last 12 months, male circumcision. | Malaria endemicity was a risk factor for HIV for men and women -- people living in areas with a high parasite rate were nearly twice as likely to be HIV positive. Probability ratio: 27% for men and 29% for women. Formerly married, urban dwelling, higher wealth, recent STI symptoms and lack of male circumcision were risk factors for HIV. |
| Cuadros et al. (2013) | Burkina Faso, Burundi, Cameroon, DRC, Ethiopia, Ghana, Guinea, Kenya, Lesotho, Liberia, Malawi, Mali, Mozambique, Rwanda, Senegal, Swaziland, Sierra Leone, Tanzania, Zambia, Zimbabwe | Identify clusters of high and low HIV in many countries using cluster detection. Evaluate association between national HIV prevalence and proportion of the population within the cluster and strength of cluster. | ArcGIS 9.2 | HIV status, stage of infection, sexual risk group. | 38 clusters of high HIV, 45 clusters of low HIV in 18 of 20 countries. Wide variation between countries in prevalence within clusters and proportion of population in cluster (p<0.001). Mathematical model of epidemic threshold showed that if countries are close to the threshold, a 10% change in risk behavior could lead to 250% increase in prevalence, but if beyond the threshold, the same increase only leads to 8% prevalence rise. Low prevalence countries had stronger clustering (higher RR) of high HIV prevalence, and high prevalence countries had stronger clustering of low HIV prevalence. |
| Cuadros et al. (2015) | Tanzania | Identify spatial associations between male circumcision (MC) and HIV using LISA and cluster detection. Compare HIV incidence rates by gender inside and outside MC cold spots. | ArcGIS, GeoDa | Sex, age, place of residence, wealth index, marital status, education, presence of genital ulcerations. | Cold spots of MC identified north and south west, closely matched LISA areas of low MC, high HIV. East of Tanzania = high MC, low HIV except Dar es Salaam (high MC, high HIV). Outside of MC cold spots, females at greater risk - RR between females and males increased from 1.25 - 1.78. Inside MC cold spots, males and females at equal risk |
| Docquier et al. (2014) | 44 countries | Identify the effect of immigration and emigration on HIV prevalence across sub-Saharan African countries using dynamic regression model | Not Reported | Past level of HIV prevalence, average level of HIV prevalence in destination countries of native emigrants from the country, average level of HIV prevalence in origin countries of foreign immigrants into the country, country and time fixed effects to control for demographic, economic, institutional and cultural characteristics | Significant effect of emigration to high-prevalence destinations on origin country HIV prevalence. No impact on country's HIV prevalence from immigration. Long-run effect of emigration accounts for more than 4% of HIV cases in 15 countries and more than 20% in 6 countries. HIV prevalence in 2000 could have been larger or smaller in different countries, depending on how emigration changed from that country during the 1990s. Spatial factors included in the SEM and SAR models are not significant. |
| Escamilla et al. (2015) | Zambia | Determine association between distance from households to clinics and uptake of PMTCT using regression analysis and kernel density estimates of uptake | ArcGIS 10.0; STATA 13.1 | Distance to clinic, maternal age, parity, education, institutional delivery, household wealth, distance to main road, maternal employment, time of initial ANC visit, knowledge of HIV, clinic attended | Areas with high-density uptake were located near health centers Distance associated with lower uptake: OR=0.90 (0.84-0.97) adjusted OR=0.90 (0.82-0.99); distance threshold of 1.9km |
| Golub et al. (2016) | Kenya | Evaluate association between distance to VMMC facility and post-operative visit attendance using regression analysis | ArcGIS 10.1, SAS 9.3 | Facility type, age, district of residence, employment status, income, live-in partner, district of clinic attended. | Greater distance associated with higher odds of non-attendance. Compared to those 0-1km from facility, those 5.01-10km had OR=0.71 (p=0.02) and those 10km away had OF=2.8 (p=0.01). Facility type, age, distance of residence confounded relationship between distance and attendance. Those attending fixed facilities significantly less likely to attend follow-up than those who lived close (not significant for mobile facilities). |
| Gonese et al. (2010) | Zimbabwe | Compare ANC surveillance with DHS data within ANC catchment area | ArcView 9.1, STATA 10.1 | Age, urban/rural, education, employment, marital status, number of living children, currently pregnant, gave birth in last 3 years, attended ANC for last birth. | ANC and DHS similar for all men and women, women aged 15-49, ANC attendees within 30km of ANC site, but ANC estimates were lower for all women within 30km of ANC site. |
| González et al. (2015) | Mozambique | Compare clusters of HIV at two time periods using cluster detection. Calculate HIV incidence estimate based on age-specific HIV prevalence at two time periods | SaTScan, STATA 12, Open Data Kit for data management | Sex, age, marital status, education, literacy, immigrants, religion | HIV incidence estimated 3.6 infections/100 person-years assuming stable epidemic conditions. Small cluster of high HIV in 2010 (0.2 sqkm), same cluster center but larger in 2012 (2.7 sqkm) with OR 2.05, p=0.002. Significantly more immigrants within the cluster than outside (56.9% vs. 39.6%, p=0.001). |
| Grabowski et al. (2014) | Uganda | Determine role of intra-community sexual networks and of viral introductions from outside of communities in continuing rural HIV transmission using spatial clustering analysis, phylogenetics, probabilistic transmission models | Not reported for spatial analysis or statistical modeling | Sex,age, marital status, self-reported recent sexual partners, locations of self-reported recent sexual partners, HIV status of self-reported recent sexual partners | 39% of new transmissions are in stable household partnerships. 62% of extra-household sexual transmissions are from outside the community. Spatial clustering outside of households weak, only distances <500m; People living with HIV-prevalent person were 3.2 times more likely to be HIV+; living with HIV-incident person 10.8 times more likely to be HIV-incident Phylogenetics: transmission frequently crosses community boundaries; Among non-household clusters, 72% crossed community boundaries |
| Houben et al. (2012) | Malawi | Track changes in travel time to the nearest clinic providing ART and clinic actually attended as services expanded between 2005 and 2009. | ArcGIS 9.3 (Spatial Analyst), R, STATA 11 | None. | Opening additional ART clinics reduced potential travel time from 83 to 43 minutes and actual travel time from 83 to 47 minutes. Proportion of patients not attending nearest clinic increased with more clinic options. Patients not attending nearest clinics more likely to transfer out, but transfers reduced when more clinics available (RR= 3.11 when 2 clinics, RR = 2.30 when 4 clinics). |
| Johnson et al. (2013) | Malawi | Evaluate the effect of Euclidean distance from patient neighborhood to clinic on timely ART initiation using regression analysis | Google Earth, SAS | Age, BMI, sex, ART eligibility criteria/WHO stage, distance from clinic, employment, ART eligible at first visit, year of enrolment, CD4 count | Multivariable regression: Distance to clinic is significant for patients of one of the clinics (Lighthouse; OR = 0.977, p<0.001), but not the other, which is located next to the city's central transportation hub. Age >40, BMI less than 18.5, and ART eligibility on the bases of CD4 count significantly reduced odds of initiation at both clinics. Some occupational effects with those not working least likely to initiate treatment. Effects diminished for those >10km away attending clinic next to transport hub. In univariate analysis, distance to clinic not significant for either clinic. |
| Kalipeni & Zulu (2008) | Continental | Interpolate international HIV prevalence maps across Africa to generate country-level estimates using IDW and kriging. Model epidemic curves for each country and project future trends | ArcGIS 9.1; EPP software package | N/A | Differences between UNAIDS estimates vs. kriging- and IDW-generated national estimates were statistically insignificant (IDW-UNAIDS p=0.6, kriging-UNAIDS p=0.7, kriging-IDW p=0.9). Confirmed with ANOVA results (p=0.6). Descriptive findings: Epicenter of HIV in Great Lakes region and Guinea-Bissau expanded in prevalence level and extent (1986-1990). Second epicenter in southern Africa grew to become more intense at 15-30% prevalence while Great Lakes nucleus became isolated pockets and new nucleus in Ethiopia/Eritrea (1990-1994). Southern African nucleus expanded, with isolated and less intense pockets in east and west Africa (1994-2003). Epidemic curve fitting: different regions have experienced different epidemics. Central and East Africa peaked first in 1985-1995. Rates low in west Africa and stabilized since early 1990s. Southern African rates much higher, peaked and levelled off in early 2000s. Models fit better in West Africa, then East Africa, then southern Africa based on log likelihood values. |
| Kandala et al. (2011) | Zambia | Compare spatial distribution of HIV prevalence over two periods using Bayesian spatial regression | BayesX 0.9 | Sex, urban/rural, age at diagnosis, region | Unadjusted marginal odds ratio: highest 2001 HIV prevalence in Lusaka (OR: 3.29, p<0.05) and Copperbelt (OR: 2.87, p<0.05). Highest 2007 HIV prevalence in Lusaka (OR: 2.62, p<0.05) and Central (OR: 2.39, p<0.05). Lowest HIV prevalence in Northwestern and Northern both years. Multivariable Bayesian geo-additive regression: Sex (women, OR 1.59 and 1.43), urban residence (OR: 2.73 and 2.35), age at HIV diagnosis, province of residence significant both years. Highest 2001 HIV in Central and Southern, highest 2007 HIV in Central and Western. Age U-shaped association, differing by sex. |
| Kandala et al. (2012) | Botswana | Describe geographic distribution of HIV prevalence and evaluate association of demographic factors using Bayesian spatial regression | BayesX 2.0.1 | Sex, age at diagnosis, urban/rural, occupation, and general district indicator | Region-specific effects showed highest marginal ORs in Selibe-Phikwe, Sowa, Francistown, lowest ORs in Kgalagadi North and Kweneng West. After accounting for spatial dependencies of districts, highest HIV prevalence included Francistown and Selibe-Phikwe, lowest prevalence was Gaborone. HIV rates higher among females and clerical/manual laborers compared with professionals. |
| Kandala et al. (2008) | Zambia | Describe geographic distribution of HIV prevalence and evaluate association of demographic factors using Bayesian spatial regression | BayesX 0.9 | Spatial autocorrelation, nonlinear age at diagnosis effects, gesexnder, urban/rural | Fixed effects: OR = 1.59 for females vs. males (p<0.001); OR = 2.73 for urban vs rural (p<0.001). Non-linear age effects stratified by gender: females peak around age 30, males peak around age 37. Crude Spatial effects: Lusaka and Copperbelt have highest marginal ORs (3.24 and 2.88). Adjusted Spatial effects: Lusaka and Copperbelt no significant effect, Southern and Central regions most at-risk |
| Kleinschmidt et al. (2007) | South Africa | Generate spatially continuous map of HIV prevalence among youth using Bayesian kriging. Identify factors associated with youth HIV risk using spatial and non-spatial regression | R | Sex, urban/rural, proportion African black, proportion unemployment, population density, proportion informal households, proportion school dropout | In multiple variable models, proportion of black Africans significant for male, female and combined analyses; proportion of area that was urban significant for male analysis and indicated that proportion urban and proportion black were mutual negative confounders in univariate analyses. Variation in HIV prevalence within provinces, with highest levels in the east of the country, particularly northwest KZN, south Mpumalanga, east FS. Dramatically higher prevalence among women than men. |
| Lakew et al. (2015) | Ethiopia | Identify HIV clusters using cluster detection. Evaluate associations between HIV and various factors using regression analysis. | SaTScan 9.1; STATA 11 | Age, urban/rural, education, wealth index, marital status, religion, region of residence, lifetime sex partners, employment | Six clusters identified, but only two statistically significant -- one large one in north-center (p<0.001, 258km radius with 2.6 RR), on small one just south of the first (p=0.003, 8.7 RR). Gambela administrative region had highest HIV prevalence, followed by Addis Ababa. Multiple regression: Being in middle (OR: 1.7), richer (OR: 2.3) and richest (OR: 4.1) wealth quintile associated with higher odds of HIV. Urban residence associated with HIV (OR: 1.8). Formerly married compared with never married (OR: 4.2). Primary (OR: 1.7) and secondary and higher (OR: 1.6) education compared to no education. Islamic adherents (OR: 0.58) less likely to have HIV than Christian. Multiple lifeltime partners (OR: 3.4) compared with one. Age groups 25-29 (OR: 1.7), 30-34 (OR: 2.0), 35-39 (OR: 2.1) compared to 45-49. Daily laborers lower odds (OR: 0.55), merchants higher odds (OR: 1.8) compared with non-working. Females higher odds than males (OR: 1.9). All significant at 5%. |
| Larmarange & Bendaud (2014) | Burkina Faso, Burundi, Côte d'Ivoire, Cameroon, Ethiopia, Gabon, Guinea, Haiti, Lesotho, Mozambique, Malawi, Rwanda, Senegal, Sierra Leone, Tanzania, Uganda, Zimbabwe | Generate HIV estimates at finer resolution sub-national level than currently calculated using prevR | R 3.1.0 | None. | Relatively widespread uncertainty in estimates, especially in Mozambique, Tanzania, Uganda. Good certainty in Malawi, Lesotho and northern Ethiopia. Quality of estimates depends on total sampling size, total number of administrative units, distribution of survey clusters across administrative division. |
| Manda et al. (2012) | South Africa | Identify and compare spatial distribution of HIV and syphilis using Bayesian spatial joint regression model | WinBUGS | HIV & syphilis prevalence, wealth index, population density | Negative correlation of syphilis and HIV: HIV prevalent in deprived and populated urban areas and in northeast of SA; syphilis prevalent in less deprived and less populated rural areas and in southwest of SA. Spatial variations after adjusting for contextual factors accounted for 71% of HIV variation. |
| McCoy et al. (2016) | Zimbabwe | Compare estimated mother-to-child-transmission and number of HIV+ infants in 2012 and 2014 | STATA 14; ArcGIS for display | Age, marital status, number of births, survey year, catchment area | MTCT not significantly different between 2012 (8.8%) and 2014 (6.7%) p=0.13. Self-reported ARV increased in women (59%-65%, p = 0.05), and infants (63%-67%, p=0.08). MTCT not uniform across catchment areas and changes in MTCT differed by catchment areas. |
| Mee et al. (2014) | South Africa | Compare spatial distribution of HIV mortality before (2007-2008) and after (2009-2010) ART provision became decentralized using cluster detection and regression analysis | STATA 10; ArcGIS 10; SaTScan 9.1; GeoDa | Sex, age at death, country of origin, year of death, residence status, education, wealth index, road distance to nearest clinic, illness duration, prior mortality rate in residence | Decrease in HIV/TB mortality rates of 30% (23-36%). Higher HIV/TB mortality rate in 2007-2008 associated with lower mortality rate ratio comparing 2009-2010 to 2007-2008 (i.e. bigger declines). Decrease of about 9% in mortality rate ratio for each unit increase in earlier mortality rate. Moran's I = -0.03 (no p-value reported). Two low-risk clusters (center of the site Aug-Dec 2010, southeast Nov 2007-Sep 2008), one high-risk cluster (low significance, lower south east June 2007-May 2008). |
| Mee et al. (2014) | South Africa | Visual analysis of traditional health use for HIV/TB deaths vs other causes and identify factors associated with traditional healthcare using regression | ArcGIS, STATA 10.0 | Age and gender standardized; distance to clinics and roads | No indication of variations in likelihood of TH use across site. TH use decreased from 77.5%-23.6% among those dying of HIV/TB. Higher TH use (multivariate analysis): Mozambicans, in each earlier 3-year period, increasing duration of illness. Road distance to nearest health center of clinic not significant in bivariate or multivariate analysis. |
| Messina et al. (2010) | Democratic Republic of Congo | Identify clusters of HIV using cluster detection, map prevalence with IDW, and identify factors associated with increased HIV risk with regression | SatScan 8.0.1, ArcGIS 9.3, STATA 10 | Age, wealth index, total lifetime number of sex partners, distance to a city, HIV prevalence within 25 km of community, sex. Not included in multivariate: education, distance from community to the nearest road and body of water, population density, HIV prevalence within 10km and 100km, conflict-related variables | Cluster detection: generally lower rates in the SW of DRC, higher in N and W of DRC. Differences between male and female distribution: NE is high for women but low for men, Southern Lubumbashi high for men but not women. Regression: men and women jointly have positive association between HIV prevalence and age, total lifetime partners, HIV prevalence within 25km of community, negative association with distance to city. Men: total lifetime partners, HIV prevalence within 25km, wealth. Women: total lifetime partners, HIV prevalence within 25km, age, distance to river, negative effect of distance to city |
| Musenge et al. (2013) | South Africa | Investigate HIV/TB mortality determinants and their spatial distribution in Agincourt and model interrelation of associated factors using Bayesian spatial regression | SQL, STATA 10 and 12.1, BayesX, R 2.12.2 | Age, sex, refugee status of parent, mother's age, household deaths, mother deceased, distance to health facility, household size, gender of household head, parity, wealth index | One main geographic hotspot in the north, two southerly areas with low mortality. Areas with greatest child HIV/TB didn't have a health facility. Greatest risk factor was maternal death. Other risk facters were lower SES, younger child, male, male-headed households. Conceptual framework map of individual, household, maternal and spatial factors. Confidence intervals narrowed after adjusting for spatial household random effects |
| Musenge et al. (2011) | South Africa | Identify risk factors for infant HIV/TB mortality using Bayesian spatial regression | R, STATA 10, BayesX | Sex, year, child's nationality, mother's nationality, mother's age at birth, maternal death, paternal death, head of household gender, antenatal visits, parity, wealth index, household deaths, household size, live births | Spatial heterogeneity in posterior risk estimates. Three "hotspots" in central, south easterly and south-westerly areas (matched another study by Sartorius 2011). Risk factors after controlling for spatiotemporal confounders: male, former foreigner, loss of mother, female head of house, socio-economic disadvantage, earlier birth, high household death, fewewr clinic visits, smaller households. Main finding: maternal orphans 3x more likely to die of HIV/TB |
| Musinguzi et al. (2009) | Uganda | Compare HIV prevalence rates between ANC surveillance sites and national population survey clusters within 30km of surveillance sites | WHO HealthMapper 4.2, ArcGIS 9.1, STATA 9.1 | Age, urban/rural, education, parity, marital status, employment, reproductive history | HIV prevalence was 5.9% [5.5-6.3%] in UHSBS and 6.0% [5.5-6.5%] in ANC data. ANC-based was higher in 15- to 19-year age group, similar for 20- to 29-year age group and lower for those aged 30 and above. ANC estimates 67% lower than UHSBS in urban areas (p=0.001) but similar in rural areas (p=0.34). Results consistent with findings from other studies on age-specific comparisons. |
| Namosha et al. (2013) | South Africa | HIV mortality cluster detection comparing pre-ART roll-out (2000-2003) and post-ART roll-out (2004-2006) | SaTScan 7.0 | Adjusted for age and sex. | Strong spatial clustering. HIV clusters 2000-2003: 1. RR = 1.46, p=0.001; 2. RR = 0.6, p=0.014; 3. RR = 0.39, p=0.003. HIV clusters 2004-2006: 1. RR = 1.51, p=0.001; 2. RR = 0.45, p=0.005 (same location as cluster 3 previously). All-cause mortality clusters essentially the same (HIV is the major cause of death). High-mortality clusters are in the peri-urban communities along the National Road. Lowest mortality in the urban township and rural areas. |
| Ngesa et al. (2014) | Kenya | Evaluate geographic distribution and association of demographic factors with HIV using Bayesian spatial regression | WinBUGS | Urban/rural, education level, marital status, age at first sex, perceived risk of HIV, circumcision status, had STI in the last 12 months, ever used condom, nonlinear effects of age, spatial effects | Significant positive effects on HIV prevalence: urban residence, primary education, lack of circumcision, having had an STI in the last 12 months, having ever used a condom. Likelihood of HIV increases with age up to 40 and then decreases. Clear evidence of spatial variation of HIV -- counties around Lake Victoria and around Mombasa have high association with HIV prevalence after controlling for other covariates. |
| Okango et al. (2016) | Kenya | Match HIV prevalence with risk factors using Bayesian spatial regression with spatially varying coefficients | R-INLA | Urban/rural, age, wealth index, media access, education, marital status, perceived risk of HIV, age at first sex, STI history, birth history, sex partners in last year, travel history | Covariates for HIV do not seem to vary significantly across space, because models with spatially varying coefficients were not noticeably different from each other, or from the models with spatially structured or unstructured random effects. Effect of education and of age at first sex on HIV prevalence was more in the North Eastern, Coastal, Southern regions and parts of Central region. Effect of number of partners in the last 1 year greater in some parts of West, Lake and Central region. Effect of marital status on HIV prevalence dominant in West and Lake regions. Visually, risk factors appear to have approximately similar distributions in their effects on HIV prevalence. Nonlinear effect of age: infection increased with age until about 30 years and then declines. |
| Okango et al. (2015) | Kenya | Evaluate geographic distribution and association of demographic factors with HIV and HSV-2 in women using Bayesian spatial regression | WinBUGS 14 | Education level, age at first sex, perceived risk, partners in the last one year, marital status, urban/rural, STI status in the last year, age | HIV & HSV-2 correlated (0.683). Spatial variation of HIV and HSV-2. Western and Lake Victoria regions had high HIV prevalence. HSV-2 prevalence highest in same regions as HIV, higher than HIV and more spread than HIV. Risk factors associated with HIV: urban residence (1.592), divorce (2.78) or widowed (4.603), no perceived risk, first sex between 12-14 compared with after 18 (1.691), no education vs higher education (2.425), STI in last 12m (1.57); Age risk was inverted U with peak around 30yrs (peak around 40 for HSV-2). |
| Palk & Blower (2015) | Lesotho | Investigate associations between partner living away from home, extramarital partners and HIV infection using kriged map and regression | R | Sex, age, marital status, HIV status, extramarital partners, residency status of partner | Approx 15% of population moved to different healthcare district between 2001-2011, ~30% moved within healthcare district. Almost half of households in interior had family members elsewhere in Lesotho, households with members in SA concentrated in border regions in southwest, southeast, northeast. Spouse living away from home not a risk factor for HIV infection. Increased odds of extramarital partner for man whose wife lived elsewhere in Lesotho (OR: 1.57, p<0.05) or in SA (OR: 2.85, p<0.001). |
| Sartorius (2013) | South Africa | Describe space-time dynamics and determinants of age-specific mortality using Bayesian spatial model | STATA 10; WinBUGS; MapInfo Professional version 9.5; Microsoft SQL | Sex, nationality, time period, season, maternal refugee status, age at pregnancy, dealth of mother during childhood, maternal education, parity, birth intervals, sibling death, household size, household deaths, household head demographics, wealth index, food security, distance to nearest health facility, ANC attendance, migration patterns, household elevation | HIV/TB leading cause of death in all age groups. Mortality increased over time and then plateaued around 2004. South-east and upper central regions are high-risk for most age groups. Identified clusters and numerous significant factors (more deaths from HIV/TB and diarrhea/malnutrition, shorter breastfeeding, deaths of previous children, mothers dying of HIV/TB, lower maternal education, cumulative household deaths, younger household heads, household heads dying, Mozambican household heads, low SES, temporary migration rates, lower proportion of secondary+ education. |
| Sartorius et al. (2011) | South Africa | Bayesian kriging of all-cause and cause-specific child mortality risk to analyse space-time variation. | SQL, STATA 10.0, OpenBUGS, R. Maps in Map Info Professional v. 9.5 | Gender, nationality, season, maternal nationality, age at pregnancy, maternal education, Mozambican family member, parity, parental death during childhood, father death before birth, household deaths, sibling death, age, birth interval, mother ANC attendance, socioeconomic quintile, food security, distance from household to clinic, household elevation | Two distinct foci of all-cause mortality in central northern and south-east areas -- south east focus grows over time, upper central focus emerges in 2000-2003. HIV/TB deaths also had foci in north central and south-east areas. HIV/TB leading cause of death. Mortality increased over the study period, particularly between 1992-2003. No association between distance to clinic and mortality. Significant risk factors: winter season, Mozambican family member, increased number of children in family, parental death during childhood (most significant: mother death when child is 1-5 years, especially of HIV/TB), father death before birth, increased number of cumulative household deaths, death of previous sibling. Protective factors: increased age, increased mother's age, mother with tertiary education, longer post-birth interval, increased mother ANC attendance, higher socioeconomic quintile. |
| Sartorius et al. (2013) | South Africa | Describe space-time dynamics and determinants of adult mortality using Bayesian spatial model | WinBUGS, STATA 12 | Age, sex, nationality, education, migrant pattern, household size, household age, household deaths, wealth index, household head demographics, distance to nearest health facility, village size, village mortality, proportion village deaths due to HIV/TB, village migration patterns | Five foci of adult mortality in central/upper central region and south-east: higher risk of communicable disease. Five villages have higher mortality risk as a function of increased distance to nearest clinic. 45% of deaths due to HIV/AIDS and TB. Mortality increased over the study period, especially from 1999-2004, then decline after 2007. Major risk factors: young adult age (15-44), male, partner death, low education, migrant status, unemployment, number adult deaths in household, female/deceased/younger adult head of household, low SES, distance from main road. Distance to healthcare only associated with higher mortality in unadjusted analysis. |
| Schaefer et al. (2017) | Zimbabwe | Compare HIV service uptake, distance to services and demographic characteristics inside and out of identified HIV Kulldorff clusters. Kriging of HIV prevalence and uptake of HIV testing and counseling (HTC). | ArcGIS 10.2.2, SaTScan 9.4.2 | Sex, age, marital status, migration, urban/rural, education, wealth index, age at first sex, number of recent partners, condum use at last sex, Euclidean and self-reported distance to clinic | Significant high HIV clusters around urban areas of Nyazura and Nyanga, which also showed highest HIV prevalence on the kriged map. Central belt of lower prevalence visible in the kriged map with a large low HIV cluster in the west-central area. No significant clusters for HIV testing uptake, but kriged map shows lower service uptake in Nyanga, Nyazura and southern area of Watsomba. One high VMMC uptake cluster near Watsomba and one low VMMC uptake cluster near Hauna (east). High HIV cluster: shorter distance to HIV testing services, lower uptake of HIV testing, longer distance to VMMC services, more recent in-migrants, more young and married people, more urban, lower odds of being in lowest wealth index tercile, younger age at first sex, higher odds of condom use. |
| Siedner et al. (2013) | Uganda | Measure correlation between four measures of distance/transport barriers, and evaluate their effects on HIV clinic attendance with regression analysis | STATA 11, R, SaTScan version 9.3, BayesX version 2.1 | Age, sex, education, CD4 cell count, wealth index | High correlation between GPS-tracked and GPS straight-line distance measures (kappa = 0.73), low correlations between GPS measures and self-reported time (kappa = 0.14-0.21) or cost (kappa = 0.10). Moderate correlation between self-reported measures (kappa = 0.35). Multivariable regression models: Missed days (log-transformed) associated with GPS tracked distance (coeff: 0.029, p<0.001) and GPS straight-line distance (coeff: 0.021, p<0.001). No association between missed days and self-reported indicators. |
| Tanser et al. (2009) | South Africa | Identify clusters of HIV prevalence using cluster detection and kernel smoothing. Compare characteristics inside and out of clusters. | SaTScan, Idrisi Andes | Education, wealth index, marital status, non-resident household members, employment | High-density settlements in south east near National Road have highest prevalence (35%); 40% of HIV+ live within 1km of National Road. 3 clusters high prevalence (RR 1.34-1.62) along the National Road; 3 clusters low prevalence (RR 0.2-0.38) rural but close to high-density settlements or deep-rural (p-values 0.001-0.017). High prevalence communities have high education, household wealth, employment, lower marriage and migrants |
| Tanser et al. (2000) | South Africa | Investigate association between HIV prevalence and distance between homestead and primary or secondary roads using regression analysis | MapInfo, Idrisi 2.0, Bmdp | Clinic attended (stratification) | Correlation coefficient between mean distance to road and HIV prevalence = 0.66 (p=0.002). Not significant for primary roads alone (p=0.45).  Variation in proximity to roads within catchment, so area-level data may obscure associations |
| The subnational estimates working group (2016) | Tanzania, Kenya, Malawi | Compare six methods for generating maps and sub-national estimates of HIV prevalence | R, BayesX | None reported. | All methods show within-country variation in HIV prevalence. Methods with additional data/covariates have more complex spatial variations (less smooth). All methods are similarly and reasonably accurate, but the Bayesian geostatistical approach is slightly better. Accuracy of predictions depends more on prevalence and survey sample than on modeling method. |
| Wabiri et al. (2016) | South Africa | Determine spatial relationship between HIV and social and demographic covariates using geographically weighted regression and OLS models | ArcGIS 10, STATA 11.0 | Urban/rural, sex, race, age, marital status, age of partner, condom use, socio-economic quintile | OLS: demographic model had no residual autocorrelation (Moran's I = 0.007, p=0.68), social model had residual autocorrelation (Moran's I = 0.22, p<0.05). Hyper-epidemic districts have very homogenous population of Blacks, high proportion single and high proportion with partner 5+ years older. GWR slightly better fit for demographic model and definitely better fit for social covariates model based on AICc and R-squared |
| Wand & Ramjee (2015) | South Africa | Investigate spatial heterogeneity of association between risk factors and HIV risk at two clinics using Bayesian spatial regression | R 2.15.1 | Education, age at first sex, lifetime partners, cohabitation with partner, transactional sex, recent sex partners. Adjusted for age. | Presented plots showing geographic variation in each risk factor, adjusting for age. Women at Umkomaas clinic less educated than those at Botha's Hill, who tended to have a higher number of sexual partners and unmarried sexual partners. Total risk score showed higher impact on Botha's Hill women than Umkomaas. Population attributable risk for any of the five factors accounted for 25% of Umkomaas and 65% of Botha's Hill seroconversions. |
| Westercamp et al. (2010) | Kenya | Describe spatial distribution of STIs and sexual behaviors among young men using cluster detection | SaTScan, ArcGIS 9.0 | Age, circumcision status, marital status, employment status, education level, sexual risk behaviors, STI at baseline, recent sexual dysfunction | No clusters found either for risk behavior or STI prevalence, conclude no association between place of residence and sexual risk behaviors |
| Wirth et al. (2015) | Botswana | Estimate effects of proximity to epidemic hotspot and age on HIV prevalence based on pairwise composite likelihood | R 3.0.2, SAS 9.3 | Age, distance from hotspot | Non-linear relationship between distance and HIV. HIV prevalence in districts in 2nd and 5th sextile from hotspot same as in 1st sextile, but significantly lower in 3rd, 4th and 6th sextile. Logistic regression ignoring correlation within and between districts provided higher estimates of the effect of proximity. HIV prevalence increased with each octile of age. |
| Yao & Murray (2014) | Mozambique | Compare current and optimized allocation of HIV testing sites to minimize population-weighted travel distances. Evaluate efficiency gains of adding or relocating services to new locations. | Shapely (open-source Python library), Gurobi (optimization software), ArcGIS | None. | Optimized clinic locations differ from existing clinics and improve average access distance by 19.2% (2006) or 24.4% (2009). Places where optimization suggests clinics should be placed do have low HIV testing rates. Analysis of relocating clinics while keeping others in same location shows that relocating just 1 clinic would increase efficiency 7% and relocating 12 clinics (optimal) would increase efficiency more than 24%. Adding clinics: add 1 clinic increases efficiency 5%, adding 11 clinics = 26.7% increase (after that efficiency continues to increase but by very little). Poorly served areas are first sites selected for expansion. |
| Yao et al. (2014) | Mozambique | Investigate changes in access to and use of HIV testing between 2006 and 2011 to assess the impact of expanded HIV services using cluster detection and regression analysis | ArcGIS 10.0, SaTScan, R | Age, education, number of children, wealth index, polygamy, HIV testing history, distance to nearest clinic, difficulty of access in rainy season | Large increase in HIV testing (17.6% to 55.4% to 78.3%), percent women within 10km of testing clinic four times higher in 2011 (84.2%) than 2006. Change in distance measures dramatic between 2006-2009, less so 2009-2011. Clusters of high and low testing changed between 2006-2009, disappeared in 2011. In 2006 and 2009, >20km from testing clinic and difficult to get to in rainy season both have significant negative effect on testing, not in 2011 (controlling for age, number of children, education, household wealth, marriage status, previous HIV testing). |
| Yao et al. (2012) | Mozambique | Investigate individual and spatial factors associated with use of SRH services using cluster analysis and detection and regression analysis | ArcGIS, R, SaTScan, GeoDa | Age, education, household wealth, village average household wealth, village women's education, village women's religious affiliation, husband migration status, distance to clinic | Low rates of utilization: 17.9% tested for HIV, 22.98km average distance to clinic with HIV testing. Significant spatial clustering for HIV testing at distances <8km or >12km (K-function), high risk and low risk clusters detected in southwest part of study area. Spatial dependence in village-level HIV testing: Moran's I is 0.164 (p<0.01), LISA detected high-high and low-low clusters. Logistic regression: distance has a significant effect (OR: 0.947 per km, p = 0.046), 5+ years of education increases use (OR: 1.58, p=0.005) and household possessions index = 2 (OR: 1.47, p=0.025) or index = 4 (OR: 1.82, p=0.016). Significant smoothing term (p<0.001) so there is residual spatial variation. |
| Zachariah et al. (2006) | Malawi | Investigate effect of cost of transport and road distance on ART initiation using regression analysis | Not Reported | Sex, age, marital status, TB type, employment, distance from ART site, cost of transport to ART site | Low acceptance of ART: only 13.6% of eligible patients initiated ART. Cost of transport significantly associated with ART acceptance (p=0.001): those who paid 50 Kwacha or less to get to the hospital were 4x more likely to initiate than those who paid 100+ Kwacha. Distance to the hospital was not significantly associated with ART |
| Zulu et al. (2014) | Malawi | Describe spatio-temporal trends and clustering of HIV using IDW, Moran's I and Getis-Ord. Identify associations between spatial trends of risk factors and of HIV using regression | ArcGIS 10.0, SPSS 20.0, GeoDa | Population density, education, migration, unemployment, population age structure, urban population, per capita consumption, HIV test history, knowledge of HIV testing, syphilis prevalence, mean distance to health facility, mean distance to health facility among people aged 30-44, mean distance to major road, mean distance to major city, mean time to all-weather road among those aged 30-44, mean time to transport among those aged 30-44, mean distance to health facility among those aged 45-59 | Overall decline in prevalence since 1999 (peak of epidemic); Southern region consistently highest prevalence; severe urban epidemic, less intense and less variable rural epidemic. Global Moran's I >0 with p<0.01 in 1994, 2003, 2005, 2007, 2010; Moran's I<0 with p <0.1 in 1995, 1996. Local Moran's I: HH clusters in southern regions fairly consistent over time; LL clusters in central region districts fairly consistent over time. OLS: mean travel time to public transport for ages 30-44 strongly associated with HIV (HH cluster in southern HIV hotspot, LL cluster in central); mean distance to main road associated with decreased HIV (LL cluster (near roads) in Blantyre, secondary coldspot in HIV hotspot); proportion ever taken HIV test positively associated with HIV prevalence; education negatively correlated with HIV prevalence. |
