# Supplementary material for: Geographic Information Systems, spatial analysis, and HIV in Africa: A scoping review
Source: PLoS One. 2019 May 3;14(5):e0216388. doi: 10.1371/journal.pone.0216388 (PMC6499437; doi:10.1371/journal.pone.0216388)
Supplement: S1 File — (DOCX) [file pone.0216388.s002.docx]

**APPENDIX S1: Search term details**

**1.1 PubMed**

"HIV Infections" [mesh] OR "HIV Infections" [all fields] OR "HIV Infection" [all fields] OR "HIV" [mesh] OR "HIV" [all fields] OR "Human Immunodeficiency Virus" [all fields] OR "Human Immunodeficiency Viruses" [all fields] OR "Acquired Immunodeficiency Syndrome" [mesh] OR "Acquired Immunodeficiency Syndromes" [all fields] OR "AIDS Virus" [all fields] OR "AIDS Viruses" [all fields] OR "Human Immunodeficiency Virus" [all fields] OR "Acquired Immune Deficiency Syndrome Virus" [all fields] OR "Acquired Immunodeficiency Syndrome Virus" [all fields] OR "Acquired Immune Deficiency Syndrome" [all fields] OR "Acquired Immuno-Deficiency Syndrome" [all fields] OR "Acquired Immuno-Deficiency Syndromes" [all fields]

AND

"Geographic Information Systems" [Mesh] OR "GIS" [all fields] OR "Geographic Information Systems" [all fields] OR "Geographic Information System" [all fields] OR "Global Positioning Systems"[all fields] OR "Global Positioning System"[all fields] OR "Spatial Analysis"[mesh] OR "Spatial Analysis"[all fields] OR "Spatial Analyses"[all fields] OR "Spatial Dependency"[all fields] OR "Spatial Dependencies"[all fields] OR "Kriging"[all fields] OR "Spatial Autocorrelation"[all fields] OR "Geocoding"[all fields] OR "Georeferencing"[all fields] OR "Point Pattern"[all fields] OR “Geographic Mapping”[all fields] OR "Spatio-Temporal Analysis"[mesh] OR "Spatio-temporal Analysis"[all fields] OR "Spatio-temporal Analyses"[all fields] OR "Spatiotemporal Analysis"[all fields] OR "Spatiotemporal Analyses"[all fields] OR "spatial clustering"[all fields] OR "spatial clusterings"[all fields] OR "spatial cluster"[all fields] OR "spatial clusters"[all fields] OR “spatial modeling”[all fields] OR “spatial pattern”[all fields] OR “spatial statistics”[all fields] OR “space-time clusters”[all fields] OR “space-time clustering”[all fields] OR “spatial epidemiology”[all fields]

**1.2 Embase**

(‘Human Immunodeficiency Virus’/exp OR 'Human Immunodeficiency Virus 1'/exp OR 'Human Immunodeficiency Virus 2'/exp OR 'Acquired Immune Deficiency Syndrome'/exp OR (‘Human Immunodeficiency Virus’ OR ‘HIV’ OR ‘HIV infections’ OR ‘HIV infection’ OR ‘Human Immunodeficiency Viruses’ OR ‘Acquired Immunodeficiency Syndrome’ OR ‘Acquired Immunodeficiency Syndromes’ OR ‘AIDS Virus’ OR ‘AIDS Viruses’ OR ‘Acquired Immune Deficiency Syndrome Virus’ OR ‘Acquired Immunodeficiency Syndrome Virus’ OR ‘Acquired Immune Deficiency Syndrome’ OR ‘Acquired Immuno-Deficiency Syndrome’ OR ‘Acquired Immuno-Deficiency Syndromes’):ab,ti)

AND

('Geographic Information System'/exp OR 'Global Positioning System'/exp OR 'Spatial Analysis'/exp OR 'Spatial Autocorrelation Analysis'/exp OR ‘Spatiotemporal Analysis'/exp OR (‘Geographic Information Systems’ OR ‘GIS’ OR ‘Geographic Information System’ OR ‘Global Positioning Systems’ OR ‘Global Positioning System’ OR ‘Spatial Analysis’ OR ‘Spatial Analyses’ OR ‘Spatial Dependency’ OR ‘Spatial Dependencies’ OR ‘Kriging’ OR ‘Spatial Autocorrelation’ OR ‘Geocoding’ OR ‘Georeferencing’ OR ‘Point Pattern’ OR ‘Geographic Mapping’ OR ‘Spatio-temporal Analysis’ OR ‘Spatio-temporal Analyses’ OR ‘Spatiotemporal Analysis’ OR ‘Spatiotemporal Analyses’ OR ‘spatial clustering’ OR ‘spatial clusterings’ OR ‘spatial cluster’ OR ‘spatial clusters’ OR ‘spatial modeling’ OR ‘spatial pattern’ OR ‘spatial statistics’ OR ‘space-time clusters’ OR ‘space-time clustering’ OR ‘spatial epidemiology’):ab,ti)

**1.3 Web of Science**

TS=("HIV Infections" OR "HIV Infection" OR "HIV" OR "Human Immunodeficiency Virus" OR "Human Immunodeficiency Viruses" OR "Acquired Immunodeficiency Syndrome" OR "Acquired Immunodeficiency Syndromes" OR "AIDS Virus" OR "AIDS Viruses" OR "Human Immunodeficiency Virus" OR "Acquired Immune Deficiency Syndrome Virus" OR "Acquired Immunodeficiency Syndrome Virus" OR "Acquired Immune Deficiency Syndrome" OR "Acquired Immuno-Deficiency Syndrome" OR "Acquired Immuno-Deficiency Syndromes")

AND

TS=("Geographic Information Systems" OR "GIS" OR "Geographic Information System" OR "Global Positioning Systems" OR "Global Positioning System" OR "Spatial Analysis" OR "Spatial Analyses" OR "Spatial Dependency" OR "Spatial Dependencies" OR "Kriging" OR "Spatial Autocorrelation" OR "Geocoding" OR "Georeferencing" OR "Point Pattern" OR “Geographic Mapping” OR "Spatio-temporal Analysis" OR "Spatio-temporal Analyses" OR "Spatiotemporal Analysis" OR "Spatiotemporal Analyses" OR "spatial clustering" OR "spatial clusterings" OR "spatial cluster" OR "spatial clusters" OR “spatial modeling” OR “spatial pattern” OR “spatial statistics” OR “space-time clusters” OR “space-time clustering” OR “spatial epidemiology”)
